# Complex Horticultural Quality Traits in Broccoli Are Illuminated by Evaluation of the Immortal BolTBDH Mapping Population

**DOI:** 10.3389/fpls.2019.01104

**Published:** 2019-09-18

**Authors:** Zachary Stansell, Mark Farnham, Thomas Björkman

**Affiliations:** ^1^School of Integrative Plant Science, Cornell University, Ithaca, NY, United States; ^2^Cornell Agritech, Cornell University, Geneva, NY, United States; ^3^USDA-ARS Vegetable Laboratory, Department of Horticulture, Charleston, SC, United States

**Keywords:** *Brassica olecarea* var. *italica*, broccoli, BolTBDH, genotype-by-sequencing, QTL mapping, complex horticultural traits, BoFLC, “TO1000”

## Abstract

Improving horticultural quality in regionally adapted broccoli (*Brassica oleracea* var. *italica*) and other *B. oleracea* crops is challenging due to complex genetic control of traits affecting morphology, development, and yield. Mapping horticultural quality traits to genomic loci is an essential step in these improvement efforts. Understanding the mechanisms underlying horticultural quality enables multi-trait marker-assisted selection for improved, resilient, and regionally adapted *B. oleracea* germplasm. The publicly-available biparental double-haploid BolTBDH mapping population (Chinese kale × broccoli; *N* = 175) was evaluated for 25 horticultural traits in six trait classes (architecture, biomass, phenology, leaf morphology, floral morphology, and head quality) by multiple quantitative trait loci mapping using 1,881 genotype-by-sequencing derived single nucleotide polymorphisms. The physical locations of 56 single and 41 epistatic quantitative trait locus (QTL) were identified. Four head quality QTL (OQ_C03@57.0, OQ_C04@33.3, OQ_CC08@25.5, and OQ_C09@49.7) explain a cumulative 81.9% of phenotypic variance in the broccoli heading phenotype, contain the *FLOWERING LOCUS C* (*FLC*) homologs Bo9g173400 and Bo9g173370, and exhibit epistatic effects. Three key genomic hotspots associated with pleiotropic control of the broccoli heading phenotype were identified. One phenology hotspot reduces days to flowering by 7.0 days and includes an additional *FLC* homolog Bo3g024250 that does not exhibit epistatic effects with the three horticultural quality hotspots. Strong candidates for other horticultural traits were identified: *BoLMI1* (Bo3g002560) associated with serrated leaf margins and leaf apex shape, *BoCCD4* (Bo3g158650) implicated in flower color, and *BoAP2* (Bo1g004960) implicated in the hooked sepal horticultural trait. The BolTBDH population provides a framework for *B. oleracea* improvement by targeting key genomic loci contributing to high horticultural quality broccoli and enabling *de novo* mapping of currently unexplored traits.

## Introduction

Improvement of broccoli and other *Brassica oleracea* vegetables (cauliflower, cabbage, kale, Gai lan, Brussels sprouts, kohlrabi, and collard) is constrained by complex interactions of many genes affecting plant architecture, developmental processes, and yield. *B. oleracea* vegetable crop groups have benefited from a number of advances in plant biotechnology, gradually increasing the overall understanding of these quality-based traits. Specifically, diversity and domestication processes (Cheng et al., 2016; Stansell et al., 2018; Yousef et al., 2018; Li et al., 2019) have been clarified, next-generation sequencing and high-quality reference genomes (Liu et al., 2014; Parkin et al., 2014; Golicz et al., 2016) have expedited discovery of molecular markers associated with key traits, and diverse mapping populations segregating for these traits have been characterized (Kianian and Quiros, 1992; Landry et al., 1992; Camargo and Osborn, 1996; Ramsay et al., 1996; Bohuon et al., 1998; Hu et al., 1998; Lan and Paterson, 2000; Sebastian et al., 2000; Lan and Paterson, 2001; Axelsson et al., 2001; Li et al., 2003; Gao et al., 2007; Brown et al., 2014; Lee et al., 2015a; Li et al., 2015). For example, projects integrating these tools such as the Eastern Broccoli Project (SCRI No. 2010-51181-21062) and the USDA Vegetable Brassica Research Project (CRIS No. 6080-21000-019-00D) have developed heat-tolerant broccoli germplasm adapted to novel environments, reducing costs and enabling more sustainable production models (Atallah et al., 2014; Farnham and Björkman, 2011).

A current limitation in *B. oleracea* vegetable crop improvement is a lack of publicly available mapping populations, constraining information integration across research programs. Attempts to unify existing maps have been limited due to variable germplasm, different marker types, and linkage group nomenclature (Hu et al., 1998). Furthermore, these populations are often difficult to maintain due to self-incompatibility (Farnham, 1998; Bohuon et al., 1998; Sebastian et al., 2000; Pink et al., 2008; Walley et al., 2012).

To address these issues, the double-haploid (DH) BolTBDH population was developed from a cross between morphologically distinct parents (*B. oleracea* var. *alboglabra* × *B. oleracea* var. *italica*) that segregates for horticultural quality traits specific to broccoli (Iniguez-Luy et al., 2009). BolTBDH offers several distinct advantages over other mapping populations: a large sample size (*N*∼175), a high degree of self-compatibility, and a short generation time. The rapid-cycling parental taxa ‘TO1000DH3’ (P_1_; var. alboglabra) is the reference organism for the *B. oleracea* v.2 genome (Parkin et al., 2014). ‘Early Big’ (P_2_; var. *italica*) has been evaluated in previous studies (Li et al., 2003; Gao et al., 2005; Tortosa et al., 2018). Moreover, both P_1_ and P_2_ and included in the *B. oleracea* pangenome (Golicz et al., 2016). Furthermore, this population has already been used to investigate the genetic control of important traits: glucosinolate content (Sotelo et al., 2014), organ-specific phenylpropanoid metabolism (Francisco et al., 2016), antioxidant content (Sotelo Pérez et al., 2014), and black rot (*Xanthomonas campestris* pv. *campestris*) resistance (Iglesias-Bernabé et al., 2019). Under standard greenhouse conditions, BolTBDH lines will typically produce self-seed without the need for hand pollinations. The work presented here increases the value of this population by generating many high-quality genome-wide SNP markers and generating robust phenotypes for 25 horticultural quality traits.

The BolTBDH population provides an unique opportunity to evaluate the genetic basis of the heading broccoli phenotype due to the marked dissimilarity between the parental lines: P_1_ is rapid-flowering (∼ 65 days to flowering) and exhibits a leafy, non-head-forming inflorescence, whereas P_2_ is relatively late-flowering (∼ 85 days to flowering) and exhibits a heading broccoli phenotype characterized by extensive meristem proliferation during floral bud development and internode elongation (Björkman and Pearson, 1998). While considerable work has investigated head formation under optimal and heat-stressed conditions (Duclos and Björkman, 2008; Farnham and Björkman, 2011; Hasan et al., 2016; Lin et al., 2019), the exact genetic basis of this phenotype remains elusive (Axelsson et al., 2001; Li et al., 2003; Gao et al., 2007; Okazaki et al., 2007; Razi et al., 2008; Matschegewski et al., 2015; Irwin et al., 2016). There is a growing consensus of the central importance of the homologous flowering timing MADS-box transcription factor *FLOWERING LOCUS C* (*BoFLC*) in regulating the reproductive transition by inhibiting downstream *BoSOC1* and *BoFT* expression; in turn, delaying a suite of floral-identity genes including *BoLFY*, *BoAP1*, and *BoCAL* (Lin et al., 2018). The genetic basis of the heading broccoli phenotype could be explained by a number of models: simple control by one or several genes, a constrictive-conditional model where multiple genetic factors must be present, or a pleiotropic model, where several key developmental genes would underlie the broccoli heading phenotype and be further modified by additional downstream factors. Under a simple control model, the heading broccoli phenotype would exhibit qualitative control by a limited number of QTL. Under a purely constrictive-conditional model, the broccoli heading phenotype would occur only when a minimum set of independent factors were present. Under a purely pleiotropic model, a small number of developmental loci or genes are implicated in heading quality traits with epistatic interactions with additional loci.

When breeding multiple quality traits in horticultural crops, it is often challenging to determine the degree that individual traits contribute to the overall quality of these crops when predictor traits are correlated. Relative-importance analyses (RIA) allows quantification of the proportional contribution of a predictor variable to the overall quality-model R^2^, considering both unique and joint contributions with other variables (Gromping, 2006) and may be used to establish breeding priorities within a horticultural context (Stansell et al., 2017). Within this study, RIA was used to evaluate the independent contribution of individual traits to overall horticultural quality.

Therefore, our main objectives were to: a) characterize the phenotypic variation of horticulturally important traits within the BolTBDH population; b) produce a reference set of robust and high-quality BolTBDH markers; c) identify optimal QTL models to best explain key horticultural quality traits important to broccoli germplasm; and d) identify which candidate *B. oleracea* developmental genes collocate with observed QTL.

## Materials and Methods

### Germplasm

The BolTBDH population was generated *via* anther culture from the parental lines ‘TO1000DH3’ DH (P_1_; *B. oleracea* var. *alboglabra*) × ‘Early Big’ DH (P_1_; *B. oleracea* var. *italica*) (Iniguez-Luy et al., 2009). Seed was provided by the USDA Vegetable Laboratory in Charleston, SC. All initial lines (*N* = 202; P_1_ and P_1_ inclusive) were increased in 2016 and closed-bud pollinations were made to verify selfing integrity. Except for P_1_ in Y_1_ due to inadequate seed quality, all lines were sown into 128 cell trays May 11 in Y_1_ and May 9 in Y_2_. Seedlings were grown in a greenhouse and transplanted into Lima silt loam fields in Geneva, NY on May 28–29 in Y_1_ and June 8–11 in Y_2_. All lines were divided into four randomized replications and transplanted onto raised beds with each plot containing 10–12 plants per genotype. Drip irrigation was applied as needed and any additional cultural practices were as previously described (Farnham and Björkman, 2011). Hourly weather data was collected locally at Cornell AgriTech (**Figure S1**).

### Traits Investigated

**Figure 1:**
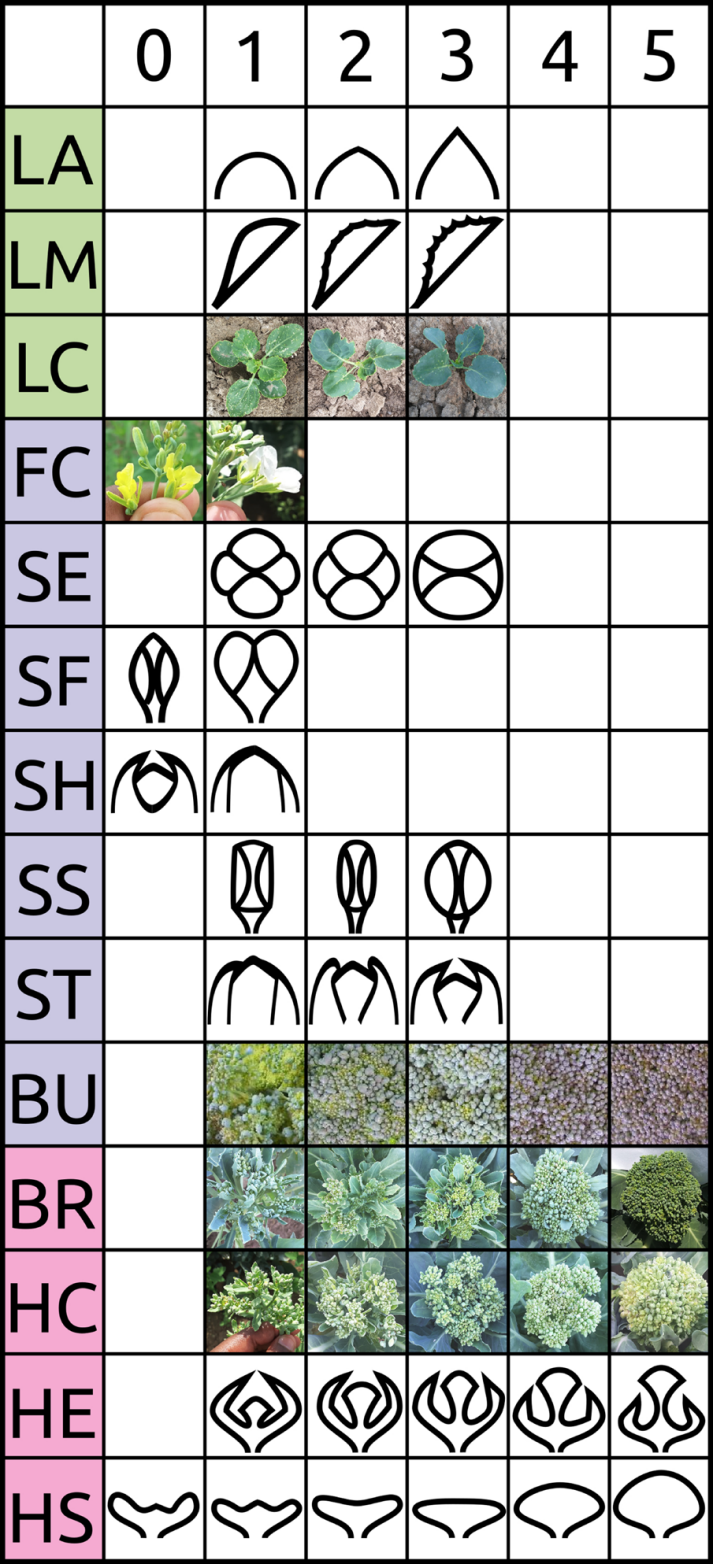
Visual scoring scale of selected traits evaluated in BolTBDH trials. See **Table 1** and Stansell et al. (2017) for additional information.

Plots were evaluated daily and deemed mature when 1/3 of the plants reached a heading or heading-equivalent stage. Traits within six classes considered important to broccoli or other *B. oleracea* crop groups were chosen: architecture, biomass, bud morphology, leaf morphology, head quality, and phenology (**Table 1**; **Figure 1**; Stansell et al. (2017)). LT was measured as the degree of lateral shoot growth. MH was evaluated as flower bud bunching before antithesis. MS was measured as above-ground biomass of a representative central plant. VG was evaluated as overall plant vigor. Leaf color/waxiness (LC) was evaluated visually. LA and LM were evaluated as the leaf-tip angle and degree of leaf margin serration. No intermediate flower color was detected so FC was scored as a binary trait. Other bud morphology traits (SE, SS, SF, ST, and SH) were evaluated visually as unopened buds at head maturity. The traits bud size (BS), bud uniformity (BU), bracting (BR), head compactness (HC), head diameter (HD), head uniformity (HU), head extension (HE), head shape (HS), overall-heading quality, and (OQ) were evaluated following standardized protocols developed by the Eastern Broccoli Project using an ordinal scale (1 = worst; 5 = best) with slight population specific modifications (e.g.: adjusting scale centering to account for smaller DH heads) (Stansell et al., 2017). Days to maturity and flowering (DM and DF) were calculated as days from sowing to head maturity and first flowering respectively. Holding ability HA was defined as DF–DM. Correlation matrices were computed between traits as well as between trial years by invoking the Spearman method with the cor() function in R v3.6.0(R-Core-Team, 2018). RIA of overall heading quality OQ was conducted with the R package ratervar (Stansell et al., 2017) using 1,000 bootstraps under the metric “lmg” by fitting the model:

(1) $$\begin{array}{l} \mathrm{OQ}∼\mathrm{LT}+\mathrm{MH}+\mathrm{MS}+\mathrm{VG}+\mathrm{LA}+\mathrm{LM}+\mathrm{LC}+\mathrm{BS}+\mathrm{BU}\\ \;+\mathrm{BR}+\mathrm{HC}+\mathrm{HD}+\mathrm{HE}+\mathrm{HS}+\mathrm{HU}+\mathrm{DF}+\mathrm{DM} \end{array}$$

**Table 1:** Trait classes and traits evaluated within BolTBDH trials with descriptions and scoring scales.

	Description	Range	Scale
*Architecture*			
**LT**	lateral shoot growth	1–5	5 = complete absence of lateral side shoots; 1 = extensive side shoots
**MH**	head bunching	0–1	1 = {HS = 2,3,4,5}; 0 = {HS = 0,1}
*Biomass*			
**MS**	above-ground biomass	(g)	Above ground biomass (g)
**VG**	plant vigor	0–5	5 = the largest plants (>0.5 m to apex); 1 = very small plants (<0.1 m to apex); 0 = died in vegetative stage
*Leaf morphology*			
**LA**	leaf apex	1–3	1 = flat; 3 = pointed
**LM**	leaf margin	1–5	1 = completely smooth; 5 = completely serrated
**LC**	leaf color/waxiness	1–3	1 = dark-green leaf color/low wax; 3 = blue-green leaf color/high wax
Bud morphology			
**FC**	flower color	0–1	0 = yellow; 1 = white
**SE**	shape of bud ends	1–3	1 = lobed; 2 = dimpled; 3 = round
**SF**	“fig” shaped bud ends	0–1	1 = fig shaped bud; 0 = not fig shaped
**SH**	hooked sepals	0–1	1 = “hooked” junction, 0 = smooth junction
**SS**	side profile of unopened flower bud	1–3	1 = straight; 2 = oval; 3 = round
**ST**	sepal junction of unopened flower buds	1–3	1 = flat junction; 2 = slight junction overlap; 3 = severe “hooked” junction
**BS**	bead size	1–5	5 = fine beads <1.1 mm diameter, 3 = medium beads (1.4–1.7 mm), 1 = extra large beads (> 2.0 mm)
**BU**	bead uniformity	1–5	5 = beads highly uniform across head surface; 3 = acceptable, but marginal uniformity of beads; 1 = highly variable bead size and appearance
*Head quality*			
**BR**	bracting	1–5	5 = head entirely free of cauline leaves bisecting curd; 3 = moderate bisection; 1 = extreme leaf bisection
**HC**	head compaction	1–5	5 = very tight floral inflorescence at head maturity; 1 = very loose inflorescence at head maturity
**HD**	head diameter	1–5	5 = largest heads observed (8–10 cm across); 1 = smallest heads (2–4 cm)
**HE**	head extension	1–5	5 = head apex is very high (e.g. >5 cm) above foliage; 3 = head apex at same position as top foliage; 1 = head apex buried deep in foliage
**HS**	head shape	0–5	5 = convex head surface; 3 = flat surface; 0 = concave head surface
**HU**	head uniformity	1–5	5 = very smooth, even surface across the head surface; 3 = acceptable uniformity across surface, moderate surface variability; 1 = highly distorted head surface
**OQ**	overall quality	1–5	5 = excellent quality; 3 = poor quality, but recognizable as broccoli; 1 = not generally recognizable as broccoli with most attributes distorted
*Phenology*			
**DM**	days to maturity	(d)	days from sowing to head maturity
**DF**	days to flowering	(d)	days from sowing to flowering
**HA**	holding	(d)	DF – DM

### Marker Development, Map Construction

DNA was extracted from young leaf tissue at the 2–3 true leaf stage, bulked from five plants and extracted according to standard protocols. GBS was accomplished at the University of Wisconsin Biotechnology Center DNA Sequencing Facility following methods of Elshire et al. (2011). Library construction occurred in 96-well plates with ApeKI digestion followed by sequencing on Illumina HiSeq 2500, producing 100-bp single-end reads. SNP production was accomplished using the TASSEL v5.2.35 GBS pipeline (Glaubitz et al., 2014): 214,757,912 raw sequence reads were initially generated and 168,722,056 (78.6%) were good barcoded reads (minimum quality score > 15; minimum K-mer count = 10; min K-mer length = 10) and reads were collapsed into 11,842,938 tags. Alignment of filtered tags were was accomplished with default settings with BWA v0.7.15 (Li, 2013) to the *B. oleracea* genome v2.1 (Parkin et al., 2014), producing 670,347 mapped tags. Initial filtering removed indels, loci with more than 10% missing data and minor SNP states. Missing data were imputed using the FSFHap plugin (Swarts et al., 2014) invoking the cluster algorithm option. Nucleotide data was recoded as ABH genotypes and heterozygous, missing, or ambiguous calls were removed. Additional quality control steps using the package rqtl (v.1.44-9; Broman et al. (2003)) were performed: taxa with > 5% missing data were removed and taxa pairs exhibiting over 95% pairwise genetic similarity were pruned. Individuals exhibiting three times more crossover events above standard deviation were removed. Ultimately, 175 DH lines were included in the final datasets. Markers exhibiting identical segregation patterns were pruned. Markers with χ^2^ −log[p.adj] > 40 segregation were removed as likely genotyping errors. Linkage disequilibrium analysis identified 87 markers that appeared to be assigned to the wrong linkage groups and these were removed. Markers were imputed using a Viterbi algorithm and genetic maps were constructed using the Kosambi mapping function (**Supplementary Data S1–S6**).

### MQM Mapping

Multiple QTL mapping (MQM) was accomplished using rqtl using the forward and backward search algorithm stepwiseqtl() by searching for QTL models with the highest penalized LOD score (Broman et al., 2003; Arends et al., 2010). Genotypes were first simulated with hidden Markov modeling (*N* = 1,000) followed by estimation of the true underlying genotype probabilities calculated across a 1 cm fixed stepwidths. Initially, 1,000 permutations of two-dimensional scans per trait were run to establish trait-specific genome-wide significance thresholds and to calculate MQM model penalties. An initial forward scan was used to determine the maximum number of QTL per trait to include in stepwiseqtl(). The normal model was invoked for stepwiseqtl() except for the trait FC which was run under a binary model. Between model selection steps in MQM analysis, QTL positions were refined using iterative maximum likelihood scanning. Additional non-parametric scans using extension of the Kruskal-Wallis tests were run as an additional confirmation step to account for non-normal distributions. Epistatic effects were identified using two-dimensional scans with the scantwo() function and chosen according to the FV_1_ model: the log_10_ likelihood of the full QTL model on chromosomes *j* and *k* was compared against a single model QTL on *j* or *k* (p_fv1_ < 0.05). QTL intervals were called by applying the function find_peaks() by choosing MQM peaks surpassing the genome-wide significance threshold (α = 0.05), estimating interval start and stop locations as 95% Bayes credible intervals (BCIs), and selecting the best adjacent markers. Percent phenotypic variance explained was calculated by

(2) $$\mathrm{PVE}=1-10^{\frac{-2*{\mathrm{LOD}}_{\mathrm{peak}}}{n}}$$

Candidates were determined by subsetting the *B. oleracea* v2.1 genome annotation by the 95% BCI followed by further extraction of *A. thaliana* BLASTP hits. Additionally, a review of candidates implicated in developmental control of the horticultural traits under consideration was followed by pan-taxonomic searches using the online tools TAIR (Berardini et al., 2015) and EnsemblePlants (Ruffier et al., 2017) to determine the physical locations of 391 *B. oleracea* homologs (**Supplementary Data S7**). These candidates were then cross-referenced against the identified QTL using a custom R script that calculated the difference in physical location of a candidate and LOD peak location, retaining only candidates located within the 95% BCI or within 1 Mbp from the LOD peak.

## Results

### Phenotyping

**Figure 2:**
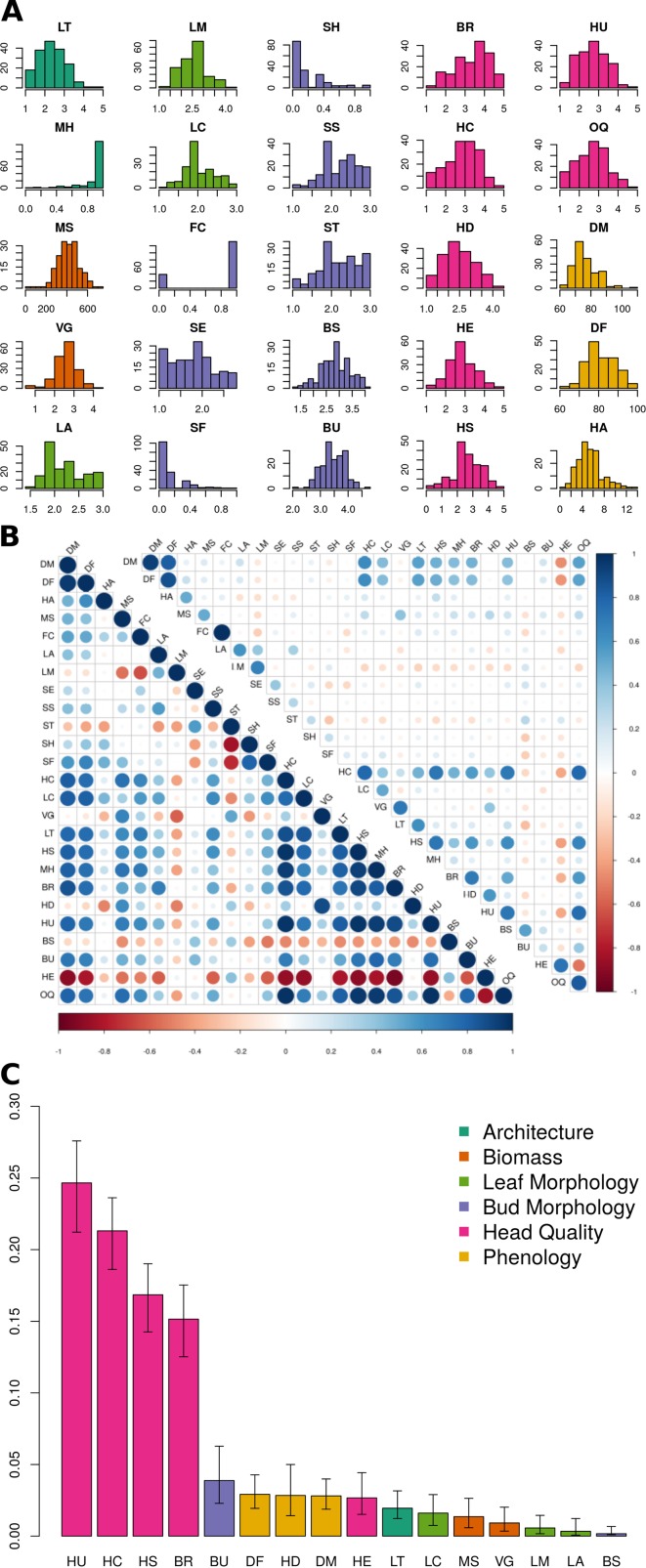
BolTBDH phenotypic analysis: **(A)** Distribution of phenotypic traits evaluated in year 1 and year 2 (Y_12_) trials. Trait class is indicated by color (architecture = dark green, biomass = orange, leaf morphology = light green, bud morphology = light blue, heading quality = pink, and phenology = yellow). **(B)** Y_12_ between-trait (bottom left) and between-year Y_1_ vs. Y_2_ (top right) Spearman correlation coefficients, with negative and positive correlations plotted in red and blue. **(C)** Percentage of Y_12_ overall quality model explained by relative importance predictors (RIA) by fitting the model [*OQ ∼ LT + MH + MS + VG + LA + LM + LC + BS + BU + BR + HC + HD + HE + HS + HU + DF + DM*] using the lmg method with the rateRvaR function raterimp() with 95% confidence intervals estimated by 1,000 permutations.

For architectural traits, suppression of lateral shoot growth (LT) was considerably higher in Y_1_ (Y_1_ = 3.1, Y_2_ = 1.8; **Figure 2A**; **Table 2**) and was moderately correlated between years (r = +0.61; **Figure 2B**). Presence of floret bunching (MH) was lower in the Y_2_ trial (0.98 vs 0.86; r = +0.22). Average above-ground biomass (MS) was 90.5 g lower in Y_2_ trials, although plant vigor (VG) was consistent between years. MS and VG were positively correlated (r = +0.57). The leaf morphology traits LA and LM exhibited moderate between-trait correlation (r = +0.45). Leaf color (LC) was moderately correlated between years (r = +0.52). Except for flower color (FC; r = +1.00), other bud morphology traits exhibited low to intermediate year-to-year correlation, ranging from “bud profile:fig” (SF; r = +0.15) to flower bud size (BS; r = +0.55).

**Table 2:** Phenotypic evaluations pooled across year 1 (Y_1_) and year 2 (Y_2_) environments ± sd; Y_1_ and Y_2_ means ±sd; (ρ) = Spearman correlations between Y_1_ and Y_2_ evaluations.

	Scale	Y_12_	Y_1_	Y_2_	ρ
*Architecture*					
**LT**	1–5	2.44 ± 0.67	3.09 ± 0.92	1.80 ± 0.54	+0.61
**MH**	0–1	0.92 ± 0.18	0.98 ± 0.11	0.86 ± 0.29	+0.22
*Biomass*					
**MS**	(g)	415 ± 105	447 ± 118	356 ± 115	+0.51
**VG**	1–5	2.67 ± 0.57	2.64 ± 0.6	2.71 ± 0.61	+0.72
*Leaf Morphology*					
**LA**	1–3	2.23 ± 0.38	2.16 ± 0.39	2.30 ± 0.45	+0.59
**LM**	1–5	2.66 ± 0.55	2.78 ± 0.66	2.63 ± 0.57	+0.67
**LC**	1–3	2.06 ± 0.39	1.96 ± 0.31	2.15 ± 0.57	+0.52
*Bud Morphology*					
**FC**	0–1	0.77 ± 0.42	0.78 ± 0.42	0.77 ± 0.42	+1.00
**SE**	1–3	1.82 ± 0.49	1.85 ± 0.57	1.75 ± 0.62	+0.38
**SF**	0–1	0.13 ± 0.21	0.17 ± 0.26	0.08 ± 0.26	+0.15
**SH**	0–1	0.20 ± 0.26	0.24 ± 0.30	0.10 ± 0.29	+0.24
**SS**	1–3	2.28 ± 0.44	2.21 ± 0.49	2.41 ± 0.56	+0.30
**ST**	1–3	2.23 ± 0.50	2.19 ± 0.58	2.34 ± 0.62	+0.26
**BS**	1–5	2.87 ± 0.57	2.84 ± 0.66	2.90 ± 0.62	+0.55
**BU**	1–5	3.48 ± 0.41	3.46 ± 0.51	3.49 ± 0.52	+0.24
*Head Quality*					
**BR**	1–5	3.43 ± 0.88	3.56 ± 1.01	3.28 ± 0.88	+0.70
**HC**	1–5	2.96 ± 0.82	3.19 ± 0.80	2.72 ± 0.92	+0.78
**HD**	1–5	2.55 ± 0.73	2.64 ± 0.86	2.45 ± 0.73	+0.58
**HE**	1–5	2.91 ± 0.68	2.95 ± 0.75	2.84 ± 0.70	+0.73
**HS**	1–5	2.72 ± 0.91	3.01 ± 0.89	2.46 ± 1.04	+0.73
**HU**	1–5	2.77 ± 0.72	2.87 ± 0.74	2.68 ± 0.79	+0.73
**OQ**	1–5	2.72 ± 0.80	2.80 ± 0.87	2.63 ± 0.79	+0.83
*Phenology*					
**DM**	(d)	76.6 ± 7.7	86.3 ± 7.2	66.6 ± 7.8	+0.93
**DF**	(d)	81.6 ± 7.4	91.3 ± 6.8	71.9 ± 8.3	+0.88
**HA**	(d)	5.45 ± 2.3	5.3 ± 1.9	5.6 ± 3.3	+0.51

All head quality traits were lower in Y_2_ when compared to Y_1_ except for bud uniformity (BU; −0.03) and head-quality traits were strongly correlated between years (r = +0.82). Head shape (HS; −0.55) and head compactness (HC; −0.47) exhibited the largest changes from Y_1_ to Y_2_ trials. Overall horticultural quality (OQ) was positively correlated with all other head quality traits such as head uniformity (HU; r = +0.94) and head shape (HS; r = 0.88) except for a negative correlation with head extension HE (r = −0.51). Days to head maturity DM (r = +0.93) and flowering DF (r = +0.88) were correlated between years, although lower in Y_2_ trials by 19.7 d and 19.4 d. DM and DF were correlated with each other (r = +0.94). Time from maturity to flowering (HA) was consistent between years (Y_1_ = 5.34 d and Y_2_ = 5.61 d) but was not strongly correlated with DM (r = +0.13) or DF (r = +0.39).

Relative importance analyses indicated that variation in the traits HU (24.7%), HC (21.3%), HS (16.4%), and BR (15.2%) explained 78.0% of the variability in overall heading quality model (**Figure 2C**, **Table S1**). Although OQ was correlated with DM (r = +0.53) and DF (r = 0.46), these traits were not strong predictors of OQ, each explaining < 3% of the variance in the RIA quality model.

### Genotyping and Mapping

**Figure 3:**
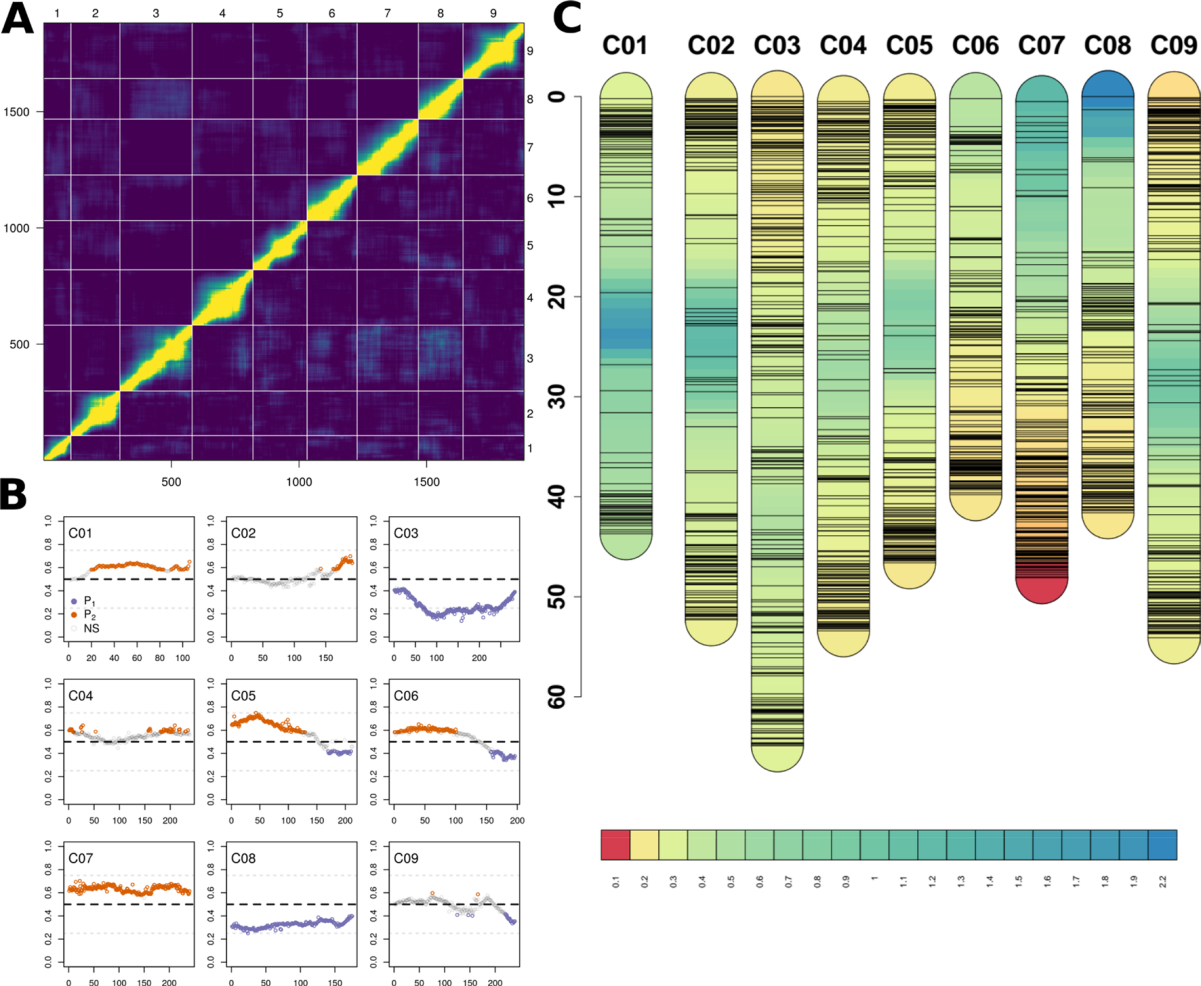
**(A)** Heatmap of relative recombination fractions (top left) and logarithm of odds scores (bottom right) comparing all pairs of the 1,881 markers (bottom and left axes) and by chromosome (top and right axes) used to construct BolTBDH genetic map. **(B)** Segregation distortion (Y-axis; *f* = P_1_ allele) observed using all markers (X-axis) disaggregatted by chromosomes (C01–C09). Markers exhibiting significant segregation distortion (χ*^2^* [p.adj] < 0.05) plotted in orange (P_1_) or purple (P_2_). Non-significant segregation distortion markers are plotted in grey. **(C)** Genetic map, marker location (cm), and marker density (cm/marker) by chromosome using all 1,881 BolTBDH markers.

Genotype-by-sequencing of all initial lines (*N* = 202) resulted in 168,722,056 quality barcoded reads distributed across 2,529,429 unique tags of which 670,347 were mapped, producing 263,998 SNPs. FSFhap imputation and filtering for minor allele frequencies, missing data, and minor SNPs states reduced this value to 15,774; decreasing percent missing data from 21.0% to 2.7% (**Figure S2**). Markers assigned to the wrong linkage groups were removed (**Figure 3A**) and 1881 high-quality, non-duplicated markers were selected and distributed with a mean coverage of 4.25 SNPs/Mbp (**Table S2**).

Lines identified as hybrids indicated by heterozygous calls, twinned lines, and crossover event outliers were removed, resulting in 175 lines included in multiple QTL mapping (**Supplementary Data S1–S3**). Deviation from the expected 1:1 segregation pattern (FDR < 0.05) was observed in 61.8% of markers and 50.9% alleles were contributed from P_1_. Several chromosomes exhibited strong segregation distortion, notably C03: 73.3% (P_1_), C08: 67.6% (P_2_), and C07: 63.3% (P_1_) (**Figure 3B**). Although segregation distortion may reduce overall QTL detection power, it does not limit detection in sufficiently dense marker sets, therefore these markers were retained.

A genetic map was constructed from 1,881 markers spanning all linkage groups (**Figures 3A–C**), with markers per chromosome ranging from 106 (C01) to 283 (C03). Total map distance was 1,060.8 cm with a mean and maximum marker spacing of 0.57 and 16.67 cm/marker. Maximum marker spacing per chromosome ranged from 2.26 cm/marker (C08) to 16.67 cm/marker (C05). Mean crossover events per double haploid were 15.0 ± 9.7; max = 61, min = 4.

### MQM Mapping

**Figure 4:**
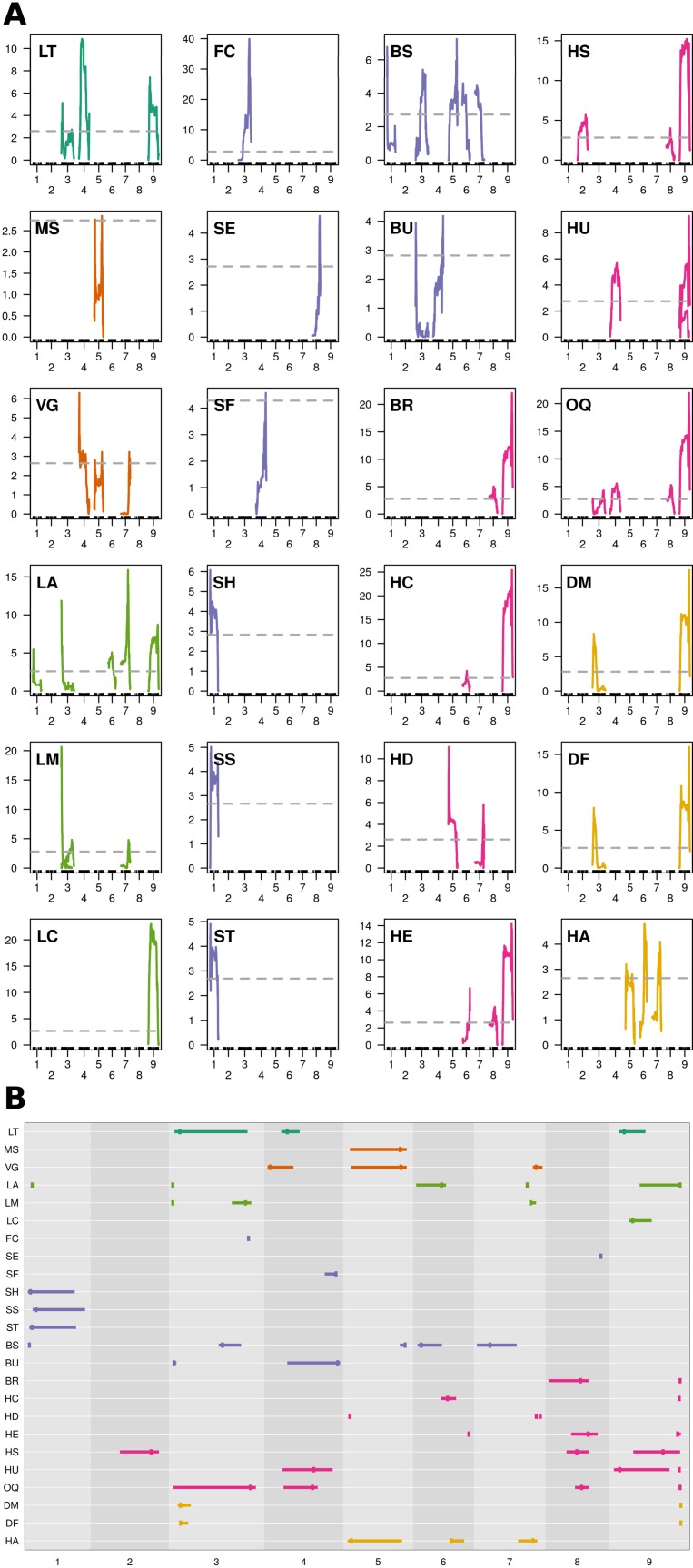
MQM QTL mapping results: **(A)** QTL plots by trait and chromosome using 1881 markers, 175 double haploid lines, and pooled phenotypes across environments (Y_12_; X-axis = chromosome, Y-axis = LOD). Horizontal, dashed, grey line = genome-wide significance threshold (α = 0.95) determined from 1,000 permutations of scantwo() for individual traits. Trait class is indicated by color (architecture = dark green, biomass = orange, leaf morphology = light green, bud morphology = light blue, heading quality = pink, and phenology = yellow). **(B)** Summarized MQM results; traits evaluated in BolTBDH printed along the Y-axis and chromosomes scaled by physical distance (Mbp) are arranged on the X-axis. MQM peak apex given by vertical bar and 95% Bayesian confidence intervals indicated by width of horizontal bar. Color schema is recycled from **(A)**.

**Figure 5:**
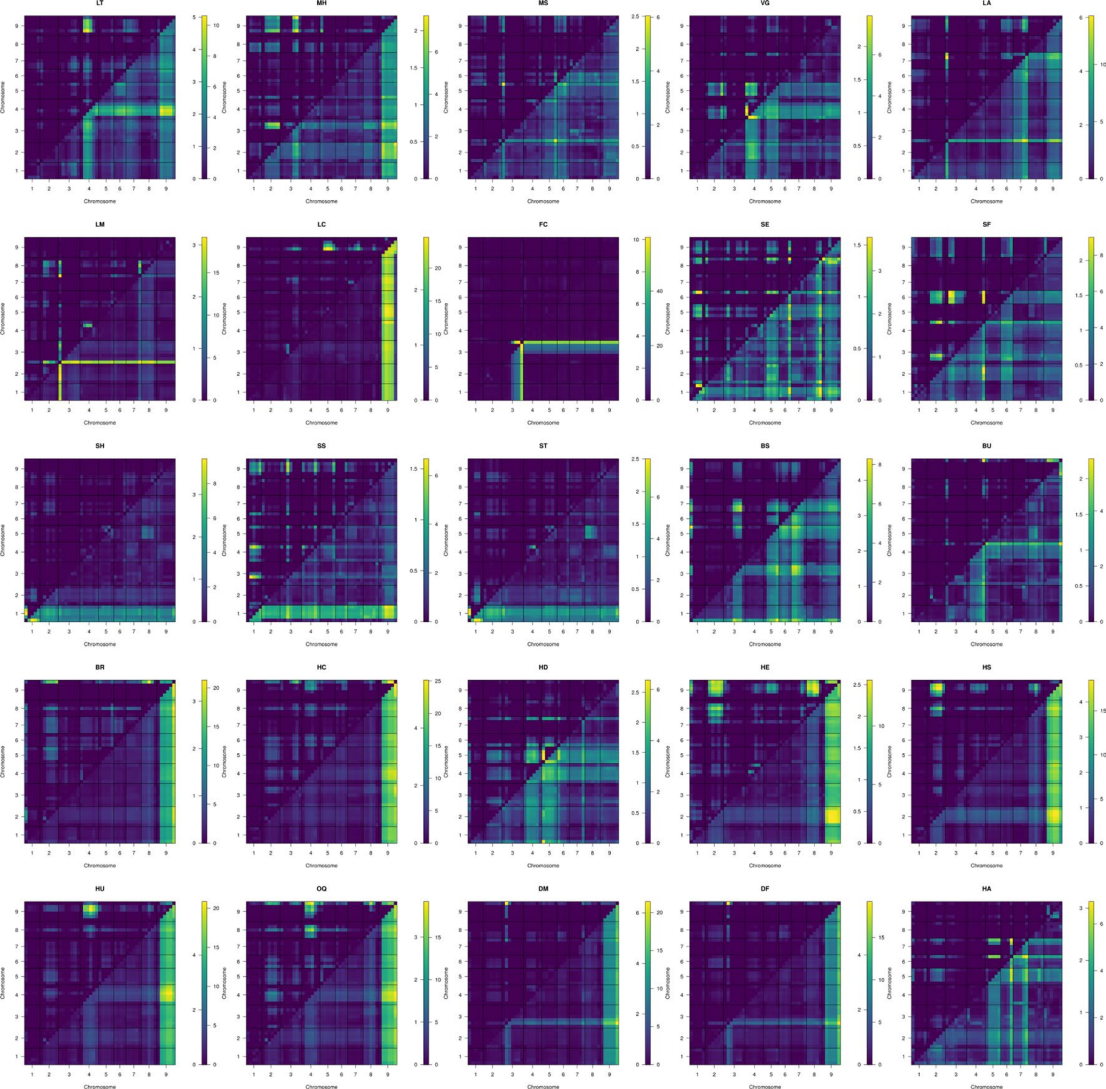
Two dimensional scans of all traits using 1,881 markers, using simulated genotype probabilities (*N* = 1000) with genotype probabilities calculated across 1 cm steps. Epistatic LOD scores are calculated the difference in the log-likelihood of the full model and the additive model for a given QTL pair and are printed above and left of trace. The full model LOD values are printed below and right of trace.

MQM identified 56 single (**Figure 4A**; **Table 3**) and 41 epistatic (**Table 5**; **Figure 5**) QTL. QTL per chromosome ranged from 1 (C02) to 12 (C09)(**Figure 4B**). LOD values ranged from 2.85 (MS_C05@39.5; PVE = 7.3) to 39.9 (FC_C03@55.7; PVE = 65.8). Bayesian confidence intervals (95% CI) ranged from 0.3 to 59.9 Mbp (mean = 16.2 Mbp), and contained on average 1902.2 coding sequences and 330.6 A. *thaliana* hits (**Table 4**). QTL per trait class ranged from 3 (biomass) to 20 (head quality). Three architecture trait QTL were identified for LT, and no MH QTL were identified. Four biomass QTL were identified, one MS and three VG. Nine leaf morphology QTL were identified: five LA, three LM, and one LC. Thirteen significant bud morphology QTL were identified: one FC, SE, SF, SH, SS, and ST, five BS, and two BU. Twenty head quality QTL were identified, including two BR, two HC, three HD, three HE, two HS, three HU, and four OQ. Seven phenology QTL were identified: two DM, two DF, and three HA. Epistatic QTL (p < 0.05) were detected in every trait class except FC, MH, and SS.

**Table 3:** Multiple mapping QTL identified in pooled Y_12_ dataset for individual traits (**Trait**) within trait classes.

	Trait	CHR	LOD	POS	ci*_low_*	ci*_high_*	MAR*	PVE	P1	P2	Δ
*Architecture*											
LT_C03@5.9	LT	3	5.1	5.9	2.0	55.0	SC3_5860860	12.69	2.64	2.37	+0.27
LT_C04@15.0	LT	4	10.8	15.0	10.6	23.9	SC4_15017345	24.96	2.68	2.17	+0.51
LT_C09@9.0	LT	9	7.4	9.0	5.1	24.4	SC9_8986159	17.81	2.66	2.19	+0.47
*Biomass*											
MS_C05@39.5	MS	5	2.8	39.5	2.9	44.2	SC5_39489450	7.26	390.38	447.64	−57.26
VG_C04@2.4	VG	4	6.3	2.4	0.7	19.4	SC4_2430671	15.34	2.86	2.42	+0.44
VG_C05@39.9	VG	5	3.2	39.9	3.9	44.2	SC5_39919197	8.18	2.54	2.84	−0.30
VG_C07@43.4	VG	7	3.2	43.4	41.0	48.0	SC7_43403602	8.19	2.80	2.49	+0.30
*Leaf Morphology*											
LA_C01@3.4	LA	1	5.5	3.4	3.0	3.9	SC1_3378511	13.46	2.15	2.36	−0.21
LA_C03@0.7	LA	3	11.9	0.7	0.0	1.0	SC3_722361	26.94	2.05	2.35	−0.31
LA_C06@18.7	LA	6	5.1	18.7	0.2	21.9	SC6_18698210	12.57	2.15	2.35	−0.20
LA_C07@37.0	LA	7	15.9	37.0	36.5	37.1	SC7_36965391	34.36	2.10	2.46	−0.37
LA_C09@49.5	LA	9	8.7	49.5	20.1	50.1	SC9_49495162	20.61	2.34	2.10	+0.24
LM_C03@0.7	LM	3	20.6	0.7	0.5	1.3	SC3_722361	42.10	2.25	2.94	−0.69
LM_C03@53.5	LM	3	4.8	53.5	43.6	57.7	SC3_53485976	11.96	2.90	2.58	+0.32
LM_C07@39.5	LM	7	4.8	39.5	38.9	43.5	SC7_39439192	11.90	2.53	2.87	−0.34
LC_C09@15.1	LC	9	23.0	15.1	12.1	28.9	SC9_15164371	45.64	2.34	1.82	+0.53
*Bud Morphology*											
FC_C03@55.7	FC	3	39.9	55.7	55.7	56.1	SC3_55671967	65.83	0.00	1.00	−1.00
SE_C08@38.0	SE	8	4.7	38.0	36.6	38.9	SC8_38021928	11.61	2.04	1.69	+0.35
SF_C04@50.3	SF	4	4.6	50.3	42.1	51.3	SC4_50348003	11.40	0.08	0.22	−0.14
SH_C01@2.1	SH	1	6.1	2.1	0.2	34.2	SC1_2101416	14.86	0.10	0.30	−0.20
SS_C01@6.1	SS	1	5.0	6.1	3.7	41.7	SC1_6098441	12.50	2.16	2.48	−0.32
ST_C01@3.2	ST	1	4.9	3.2	1.3	35.2	SC1_3205069	12.18	2.37	2.02	+0.36
BS_C01@1.3	BS	1	6.8	1.3	0.8	1.9	SC1_1324660	16.49	3.06	2.67	+0.39
BS_C03@36.8	BS	3	5.4	36.8	34.0	50.3	SC3_36786929	13.40	3.13	2.78	+0.36
BS_C05@43.0	BS	5	7.2	43.0	39.1	43.4	SC5_42975016	17.53	3.04	2.69	+0.35
BS_C06@3.8	BS	6	4.6	3.8	1.2	18.9	SC6_3829212	11.53	2.74	3.04	−0.30
BS_C07@9.9	BS	7	4.5	9.9	0.5	29.4	SC7_9938249	11.18	3.00	2.65	+0.35
BU_C03@1.7	BU	3	4.0	1.7	1.3	3.4	SC3_1653377	10.00	3.63	3.37	+0.25
BU_C04@51.5	BU	4	4.2	51.5	15.0	53.4	SC4_51533618	10.54	3.59	3.33	+0.26
*Head Quality*											
BR_C08@23.2	BR	8	5.0	23.2	0.0	29.0	SC8_23202902	12.54	3.82	3.24	+0.57
BR_C09@49.5	BR	9	22.1	49.5	48.8	49.6	SC9_49467903	44.46	3.95	2.82	+1.14
HC_C06@23.0	HC	6	4.2	23.0	18.2	29.2	SC6_23004082	10.66	3.11	2.71	+0.41
HC_C09@48.8	HC	9	25.4	48.8	48.8	49.5	SC9_48825632	49.19	3.48	2.37	+1.12
HD_C05@2.9	HD	5	11.1	2.9	2.6	3.3	SC5_2892857	25.57	2.40	2.88	−0.48
HD_C07@43.6	HD	7	5.8	43.6	43.1	44.5	SC7_43553366	14.39	2.70	2.25	+0.45
HD_C07@46.5	HD	7	3.9	46.5	46.4	47.6	SC7_46405906	9.97	2.72	2.24	+0.47
HE_C06@38.5	HE	6	6.7	38.5	38.2	38.9	SC6_38501911	16.28	3.12	2.78	+0.35
HE_C08@28.8	HE	8	4.5	28.8	16.4	35.7	SC8_28751686	11.24	2.60	3.04	−0.44
HE_C09@47.7	HE	9	14.2	47.7	47.1	50.1	SC9_47686952	31.48	2.57	3.23	−0.66
HS_C02@41.7	HS	2	5.7	41.7	19.1	47.5	SC2_41701063	14.05	3.00	2.44	+0.57
HS_C08@20.6	HS	8	4.0	20.6	13.0	29.0	SC8_20577182	10.11	3.17	2.54	+0.63
HS_C09@37.1	HS	9	15.2	37.1	15.5	49.5	SC9_37685257	33.30	3.27	2.27	+1.01
HU_C04@34.2	HU	4	5.7	34.2	11.5	47.9	SC4_34234727	14.03	2.98	2.52	+0.47
HU_C09@5.6	HU	9	4.0	5.6	1.4	41.8	SC9_5621807	10.03	3.08	2.34	+0.74
HU_C09@48.8	HU	9	9.3	48.8	48.8	49.5	SC9_48825632	21.92	3.19	2.30	+0.89
OQ_C03@57.0	OQ	3	4.3	57.0	1.1	61.0	SC3_56998411	10.79	2.47	2.78	−0.32
OQ_C04@33.3	OQ	4	5.5	33.3	12.3	37.0	SC4_33280409	13.73	2.95	2.43	+0.51
OQ_C08@24.0	OQ	8	5.3	24.0	19.2	29.0	SC8_23363239	13.08	3.04	2.56	+0.48
OQ_C09@49.5	OQ	9	21.9	49.5	48.8	49.5	SC9_49484618	44.24	3.17	2.17	+1.00
*Phenology*											
DM_C03@6.4	DM	3	8.3	6.4	4.3	13.9	SC3_6383243	19.87	81.96	74.71	+7.25
DM_C09@50.0	DM	9	17.6	50.0	49.5	50.1	SC9_50021553	37.42	80.46	71.62	+8.84
DF_C03@6.4	DF	3	8.0	6.4	5.5	12	SC3_6383243	19.2	86.78	79.81	+6.97
DF_C09@50.0	DF	9	16.0	50.0	50.0	50.1	SC9_50021553	34.84	85.22	76.97	+8.26
HA_C05@4.0	HA	5	3.2	4.0	1.3	40.7	SC5_4028759	8.22	5.89	4.65	+1.24
HA_C06@25.9	HA	6	4.8	25.9	24.8	35.0	SC6_25942835	12.07	6.11	4.53	+1.58
HA_C07@41.2	HA	7	4.1	41.2	30.4	44.3	SC7_41180016	10.40	6.04	4.68	+1.36

Chromosome (**CHR**), logarithm of odds (**LOD**), and physical position (**POS**; Mbp) of peak maxima with lower (**ci**_low_) and upper (**ci**_low_) bounds of 95% Bayesian confidence intervals. The best SNP marker (MAR*) for a given QTL and percent phenotypic variance explained (**PVE**) by QTL as calculated by is given. Parental means for “TO1000” (**P**_1_) and “Early Big” (**P**_2_), with QTL effects as (Δ) = **P**_2_−**P**_1_.

**Table 4:** Key genomic regions (**hotspot**) associated with multiple traits within BolTBDH multiple QTL mapping, identified by chromosome (**chr**), and interval (**start** and **stop**; Mbp) and single trait QTL identified within the interval.

Hotspot	chr	start	stop	QTL
**Bud** **_1_**	1	1.3	6.1	BS_C01@1.3, SH_C01@2.1, ST_C01@3.2, LA_C01@3.4, SS_C01@6.1
**Lea** **_3_**	3	0.7	1.7	LA_C03@0.7, LM_C03@0.7, BU_C03@1.7
**Phe** **_3_**	3	6.4	6.4	DM_C03@6.4, DF_C03@6.4
**HQ** **_4_**	4	33.3	34.2	OQ_C04@33.3, HU_C04@34.2
**Bud** **_4_**	4	50.3	51.5	SF_C04@50.3, BU_C04@51.5
**Bio** **_5_**	5	39.5	39.9	MS_C05@39.5, VG_C05@39.9
**Lea** **_7_**	7	37.0	39.5	LA_C07@37.0, LM_C07@39.5
**Bio** **_7_**	7	43.4	43.6	VG_C07@43.4, HD_C07@43.6
**HQ** **_8_**	8	20.6	28.8	HS_C08@20.6, BR_C08@23.2, OQ_C08@24.0, HE_C08@28.8
**HQ** **_9_**	9	47.7	50.0	HE_C09@47.7, HC_C09@48.8, HU_C09@48.8, LA_C09@49.5, BR_C09@49.5, OQ_C09@49.5, DM_C09@50.0, DF_C09@50.0

Optimal MQM models were determined for all traits ranging in complexity from FC: FC ∼ 3@55.7 to LA ∼ 1@3.4 + 3@0.7 + 6@18.7 + 7@37.0 + [3@0.0 × 9@48.5] + [3@0.0 × 7@36.6] + [7@36.9 x 9@24.4] + [1@2.6 x 9@49.5] + [6@18.7 × 7@36.7] + [1@2.6 × 3@0.0] + [2@3.1 × 8.5] (**Table 5**).

**Table 5:** Optimal MQM models for traits evaluated in BolTBDH using 1,881 markers within Y_12_ dataset. Epistatic interactions [QTL_1_ × QTL_2_] calculated using penalties assigned from 1,000 permutations of scantwo() and included when p_fv1_ < 0.05.

	Model
*Architecture*	
**LT**	∼ 3@5.9 + 4@15.0 + 9@9.0 + [4@15.0 × 9@9.0] + [5@39.5 × 6@28.3]
**MH**	NA
*Biomass*	
**MS**	∼ 5@39.5 + [3@0 × 5@40.3]
**VG**	∼ 4@2.4 + 5@39.9 + 7@43.4 + [4@0.5 × 4@20.5]
*Leaf morphology*	
**LA**	∼ 1@3.4 + 3@0.7 + 6@18.7 + 7@37.0 + [3@0 × 9@48.5] + [3@0 × 7@36.6] + [7@36.9 × 9@24.4] + [1@2.6 × 9@49.5] + [6@18.7 × 7@36.7] + [1@2.6 × 3@0.0] + [2@3.1 × 2@28.5]
**LM**	∼ 3@0.7 + 3@53.5 + 7@39.5 + [3@0.7 × 3@53.5] + [2@5.5 × 3@0.7]
**LC**	∼ 9@15.1 + [5@3.8 × 9@15.1]
*Bud morphology*	
**FC**	∼ 3@55.7
**SE**	∼ 8@38.0 + [5@7.7 × 5@21.3]
**SF**	∼ 4@50.3 + [2@40.2 × 4@50.5]
**SH**	∼ 1@2.1 + [1@0.2 × 1@20.2]
**SS**	∼ 1@6.1
**ST**	∼ 1@3.2 + [5@7.5 × 5@22.9]
**BS**	∼ 1@1.3 + 3@36.8 +5@43.0 + 6@3.8 + [1@1.3 × 5@39.5] +[5@43 × 6@3.9]
**BU**	∼ 3@1.7 + 4@51.5
*Head morphology*	
**BR**	∼ 8@23.2 + 9@49.5 + [8@21.4 × 9@49.5]
**HC**	∼ 6@23.0 + 9@48.8 + [3@53.6 × 9@49.5] + [5@1.4 × 9@49.5] + [3@53.6 × 8@22.8]
**HD**	∼ 5@2.9 + 7@43.6/46.5 + [5@2.9 × 7@44.1] + [1@1.1 × 5@1.5]
**HE**	∼ 6@38.5 + 8@28.8 + 9@47.7 + [6@38.5 × 9@47.7] + [2@5.5 × 9@47.7]
**HS**	∼ 2@41.7 + 8@20.6 + 9@37.1 + [2@41.7 × 9@37.1] + [8@20.9 × 9@48.8] + [4@51.8 × 9@39]
**HU**	∼ 4@34.2 + 9@5.6 + 9@48.8 + [4@34.2 × 9@49.5]
**OQ**	∼ 3@57.0 + 4@33.3 + 8@24.0 + 9@49.5 + [8@25.5 × 9@49.5] + [5@1.3 × 9@49.5] + [3@53.6 × 8@23.3]
*Phenology*	
**DM**	∼ 3@6.4 + 9@50.0 + [3@6.4 × 9@50.0] + [3@6.4 × 7@42.0]
**DF**	∼ 3@6.4 + 9@50.0 + [3@6.4 × 9@50.0]
**HA**	∼ 5@4.0 + 6@25.9 + 7@41.2 + [9@9.5 × 9@10.7] + [5@36.6 × 5@37.3]

Ten QTL hotspots appearing to harbor QTL across multiple traits were identified: two biomass related (Bio_5_ and Bio_7_), four morphology related (Bud_1_, Lea_3_, Bud_4_, and Lea_7_), three heading quality related (HQ_4_, HQ_8_, and HQ_9_), and one phenology related (Phe_3_) (**Table 4**).

## Discussion

### Phenotyping

Field evaluations were conducted in growing seasons that differed somewhat in temperature stress (**Figure S1**). In year two (Y_2_), a strong heat wave in the first half of July (mean high = 29.5°C) coincided with the reproductive transition. Horticultural quality scores were lower in Y_2_ trails, likely due to less optimal temperatures during the transition to flowering (**Table 2**) [e.g.: suppression of lateral shoot growth (LT) was evaluated 1.3 points lower in Y_2_ trials].

### Genotyping and Mapping

The GBS markers (**Figure 3A** and **Figure S2**) generated were high quality with less than 5% missing data and distributed across all chromosomes with a mean coverage 1.77 markers/cM (**Figure 3C**; **Table S2**; **Figure S3**), a six-fold improvement in marker coverage from previous BolTBDH maps, which relied upon approximately 300 SSR and RFLP markers (Sotelo Pérez et al., 2014; Francisco et al., 2016). Segregation distortion was prevalent but chromosome-specific (C03, C07, C08; **Figure 3B**).

### MQM Analysis

#### Architecture

Excessive lateral side-shoot growth is a horticultural defect that increases harvest costs and reduces yield. Although improved broccoli F_1_ hybrids typically exhibit strong apical dominance, variability in lateral side-shoot growth occurs among genotypes, typically under environmentally stressed conditions (Potters et al., 2007). In a bulk-segregant analysis of shoot branching in *B. juncea*, Muntha et al. (2018) identified *BjPAT1* and its signal integrator *BjBRC1* as branching candidates. The *BoPAT1* or *BoBRC1* homologs did not collocate with the LT BCI identified in BolTBDH. Tyagi et al. (2019) determined that mutations in *BjSOC1* may influence degree of lateral branching. A *BoSOC1* ortholog (Bo3g038880) was identified within the 95% LT_C03@5.9 BCI, although this candidate was located >9.5 Mbp from the LOD peak, and is not likely involved in the LT phenotype response observed in BolTBDH. He et al. (2017) conducted GWA and QTL mapping of lateral branching in *B. napus* and identified *BnaC03g63480D* as a branching number candidate. The best *BnaC03g63480D* ortholog, Bo3g159770, was nominally located within the LT_C03@5.9 BCI, but is not considered a likely candidate within this population. Additional candidates associated with meristem identity and fate identified within LT QTL are listed (**Table 6**).

**Table 6:** For traits (Trait) evaluated within BolTBDH, the MQM QTL (**QTL**) determined by 95% Bayesian confidence intervals or ± 1 Mbp from LOD peak is (**Candidate**) intersected with homologous candidates (**Homolog**) identified by literature review/TAIR/EnsemblePlants.

Trait	QTL	Homolog	Candidate	Reference
**LT**	LT_C03@5.9	*VIN3*	Bo3g019340	(Lin et al., 2005; Matschegewski et al., 2015; Ridge et al., 2015; Shea et al., 2018)
	LT_C04@15	*TSF*	Bo4g061100	(Shen et al., 2018)
	LT_C09@9.0	*GRF6*	Bo9g018730	(Wang et al., 2010; Leijten et al., 2018)
		*FD*	Bo9g024710	(Siriwardana and Lamb, 2012; Matschegewski et al., 2015; Leijten et al., 2018)
**MA**	MS_C05@39.5	*BRC1*	Bo1g117490	(Muntha et al., 2018; Shah et al., 2018; Lu et al., 2019)
**VG**	VG_C04@2.4	*SPL9*	Bo4g015800	(Siriwardana and Lamb, 2012; Leijten et al., 2018)
		*FPA*	Bo4g019780	(Lin et al., 2005; Leijten et al., 2018; Shea et al., 2018)
		*ARL*	Bo4g021250	(Wang et al., 2010)
		*SOC1*	Bo4g024850	(Lin et al., 2005; Siriwardana and Lamb, 2012; Matschegewski et al., 2015; Ridge et al., 2015; Leijten et al., 2018; Lin et al., 2018)
		*ELF4*	Bo(4g025620/4g025580)	(Leijten et al., 2018)
	VG_C05@39.9	*REM1*	Bo(5g136900/5g136880)	(Duclos and Björkman, 2008)
		*BRC1*	Bo5g117410	(Muntha et al., 2018; Shah et al., 2018; Lu et al., 2019)
	VG_C07@43.4	*REM1*	Bo(7g115340/7g115310)	(Duclos and Björkman, 2008)
		*VIN3*	Bo7g114310	(Lin et al., 2005; Matschegewski et al., 2015; Ridge et al., 2015; Shea et al., 2018)
**LA**	LA_C03@0.7	*LMI1*	Bo3g002560	(Siriwardana and Lamb, 2012; Ni et al., 2017)
	LA_C06@18.7	*GCT*	Bo6g051250	(Gillmor et al., 2014)
	LA_C07@36.6	*GIF1*	Bo7g093130	(Wang et al., 2010; Siriwardana and Lamb, 2012)
**LM**	LM_C03@0.7	*LMI1*	Bo3g002560	(Siriwardana and Lamb, 2012; Ni et al., 2017)
**LC**	LC_C09@15.1	*MAH1*	Bo9g053360 Bo9g053340 Bo9g053260 Bo9g053220 Bo9g053170	(Lee et al., 2015b)
**FC**	FC_C03@55.7	*CCD4*	Bo3g158650	(Zhang et al., 2015)
**SE**	SE_C08@36.8	*RAV1*	Bo8g107500	(Siriwardana and Lamb, 2012)
**SH**	SH_C01@2.1	*AP2*	Bo1g004960	(Siriwardana and Lamb, 2012; Matschegewski et al., 2015; Branham et al., 2017; Zhang et al., 2018)
		*AG*	Bo1g020110	(Siriwardana and Lamb, 2012; Lin et al., 2019)
**SS**	SS_C01@6.1	*AP2*	Bo1g004960	(Siriwardana and Lamb, 2012; Matschegewski et al., 2015; Branham et al., 2017; Zhang et al., 2018)
		*AG*	Bo1g020110	(Siriwardana and Lamb, 2012; Lin et al., 2019)
**ST**	ST_C01@3.2	*AP2*	Bo1g004960	(Siriwardana and Lamb, 2012; Matschegewski et al., 2015; Branham et al., 2017; Zhang et al., 2018)
		AG	Bo1g020110	(Siriwardana and Lamb, 2012; Lin et al., 2019)
**BS**	BS_C01@1.3	*AP2*	Bo1g004960	(Siriwardana and Lamb, 2012; Matschegewski et al., 2015; Branham et al., 2017; Zhang et al., 2018)
		*FD*	Bo1g006110	(Siriwardana and Lamb, 2012; Matschegewski et al., 2015; Leijten et al., 2018)
		*ROT3*	Bo1g005700	(Wang et al., 2010)
	BS_C03@36.8	*LFY*	Bo3g109270	(Duclos and Björkman, 2008; Siriwardana and Lamb, 2012; Leijten et al., 2018; Sun et al., 2018; Lin et al., 2019)
		*FAS2*	Bo3g101080	(Boseon et al., 2019)
		*GRF6*	Bo3g099210	(Wang et al., 2010; Leijten et al., 2018)
	BS_C05@43.0	*REM1*	Bo(5g136900/5g136880)	(Duclos and Björkman, 2008)
	BS C07@9.9	*REM1*	Bo(7g054150/7g054160)	(Duclos and Björkman, 2008)
**BU**	BU_C03@1.7	*LMI1*	Bo3g002560	(Siriwardana and Lamb, 2012; Ni et al., 2017)
		*FY*	Bo3g009200	(Lin et al., 2005; Okazaki et al., 2007; Siriwardana and Lamb, 2012; Lin et al., 2019)
		*REM1*	Bo3g007530	(Duclos and Björkman, 2008)
		*EMF1*	Bo3g007090	(Okazaki et al., 2007; Siriwardana and Lamb, 2012)
		*FLC*	Bo3g005470	(Okazaki et al., 2007; Matschegewski et al., 2015; Ridge et al., 2015; Hasan et al., 2016; Lin et al., 2018; Lin et al., 2019)
**BR**	*BR*_C08@23.2	CDF5	Bo8g076530	(Shen et al., 2018)
		*REM1*	Bo8g071450	(Duclos and Björkman, 2008)
	*BR*_C09@49.5	FLC	Bo(9g173400/9g173370)	(Okazaki et al., 2007; Matschegewski et al., 2015; Ridge et al., 2015; Hasan et al., 2016; Irwin et al., 2016; Lin et al., 2018; Lin et al., 2019)
		*GRF8*	Bo9g172070	(Wang et al., 2010)
		*CO*	Bo9g163730	(Osborn et al., 1997; Bohuon et al., 1998; Rae et al., 1999; Axelsson et al., 2001; Okazaki et al., 2007; Irwin et al., 2016; Leijten et al., 2018)
		*COL1*	Bo9g163720	(Lagercrantz et al., 2002)
**HC**	HC C06@23.0	*ARL*	Bo6g076730	(Wang et al., 2010)
		*LMI2*	Bo6g077600	(Siriwardana and Lamb, 2012)
	HC_C09@48.8	*SEP1*	Bo9g163790	(Varaud et al., 2011; Siriwardana and Lamb, 2012; Zhang et al., 2018)
		*CO*	Bo9g163730	(Osborn et al., 1997; Bohuon et al., 1998; Rae et al., 1999; Axelsson et al., 2001; Okazaki et al., 2007; Irwin et al., 2016; Leijten et al., 2018)
		*COL1*	Bo9g163720	(Lagercrantz et al., 2002)
		*FLC*	Bo(9g173400/9g173370)	(Okazaki et al., 2007; Matschegewski et al., 2015; Ridge et al., 2015; Hasan et al., 2016; Irwin et al., 2016; Lin et al., 2018; Lin et al., 2019)
		*GRF8*	Bo9g172070	(Wang et al., 2010)
**HD**	HD_C05@2.9	*BIGPETAL*	Bo5g010880	(Wang et al., 2010; Varaud et al., 2011)
	HD_C07@43.6/46.5	*REM1*	Bo(7g115340/7g115310)	(Duclos and Björkman, 2008)
		*FD*	Bo7g117660	(Siriwardana and Lamb, 2012; Matschegewski et al., 2015; Leijten et al., 2018)
		*AP2*	Bo7g118400	(Siriwardana and Lamb, 2012; Matschegewski et al., 2015; Branham et al., 2017; Zhang et al., 2018)
		*ROT3*	Bo7g117920	(Wang et al., 2010)
**HE**	HE_C06@38.5	*TSF*	Bo6g120900	(Shen et al., 2018)
	HE_C08@28.	*AP3*	Bo9g161800	(Varaud et al., 2011; Siriwardana and Lamb, 2012; Zhang et al., 2018)
	HE C09@47.7	*CO*	Bo9g163730	(Osborn et al., 1997; Bohuon et al., 1998; Rae et al., 1999; Axelsson et al., 2001; Okazaki et al., 2007; Irwin et al., 2016; Leijten et al., 2018)
		*COL1*	Bo9g163720	(Lagercrantz et al., 2002)
		*SEP1*	Bo9g163790	(Varaud et al., 2011; Siriwardana and Lamb, 2012; Zhang et al., 2018)
		*TFL2*	Bo9g159960	(Okazaki et al., 2007; Duclos and Björkman, 2008; Siriwardana and Lamb, 2012; Schiessl et al., 2015; Branham et al., 2017; Leijten et al., 2018)
**HS**	*HS* C02@41.7	UFO	Bo(2g121010/2g121010)	(Duclos and Björkman, 2008; Siriwardana and Lamb, 2012)
	*HS* C08@20.6	REM1	Bo8g071450	(Duclos and Björkman, 2008)
		*ARF5*	Bo8g069820	(Siriwardana and Lamb, 2012; Zheng et al., 2019)
**HU**	HU_C04@34.2	*AP3*	Bo4g120010	(Varaud et al., 2011; Siriwardana and Lamb, 2012; Zhang et al., 2018)
		*REM1*	Bo4g140670	(Duclos and Björkman, 2008)
		*SPL15*	Bo4g109710	(Siriwardana and Lamb, 2012; Leijten et al., 2018)
	HU_C09@5.6	*FPF1*	Bo9g011550	(Schiessl et al., 2015)
		*CO*	Bo9g011530	(Osborn et al., 1997; Bohuon et al., 1998; Rae et al., 1999; Axelsson et al., 2001; Okazaki et al., 2007; Irwin et al., 2016; Leijten et al., 2018)
		*GIF1*	Bo9g010000	(Wang et al., 2010; Siriwardana and Lamb, 2012)
	HU_C09@48.8	CO	Bo9g163730	(Osborn et al., 1997; Bohuon et al., 1998; Rae et al., 1999; Axelsson et al., 2001; Okazaki et al., 2007; Irwin et al., 2016; Leijten et al., 2018)
		*COL1*	Bo9g163720	(Lagercrantz et al., 2002)
		*TFL2*	Bo9g159960	(Okazaki et al., 2007; Duclos and Björkman, 2008; Siriwardana and Lamb, 2012; Schiessl et al., 2015; Branham et al., 2017; Leijten et al., 2018)
**OQ**	OQ_C03@57	*AG*	Bo3g157480	(Siriwardana and Lamb, 2012; Lin et al., 2019)
		FD	Bo3g156810	(Siriwardana and Lamb, 2012; Matschegewski et al., 2015; Leijten et al., 2018)
	OQ_C04@33.3	*AP3*	Bo4g120010	(Varaud et al., 2011; Siriwardana and Lamb, 2012; Zhang et al., 2018)
	OQ_C08@24	*CDF5*	Bo8g076530	(Shen et al., 2018)
		*REM1*	Bo8g071450	(Duclos and Björkman, 2008)
	OQ_C09@49.7	*FLC*	Bo(9g173400/9g173370)	(Okazaki et al., 2007; Matschegewski et al., 2015; Ridge et al., 2015; Hasan et al., 2016; Irwin et al., 2016; Lin et al., 2018; Lin et al., 2019)
		*GRF8*	Bo9g172070	(Wang et al., 2010)
		*CO*	Bo9g163730	(Osborn et al., 1997; Bohuon et al., 1998; Rae et al., 1999; Axelsson et al., 2001; Okazaki et al., 2007; Irwin et al., 2016; Leijten et al., 2018)
		*COL1*	Bo9g163720	(Lagercrantz et al., 2002)
**DM**	DM C03@6.4	*FLC*	Bo3g024250	(Okazaki et al., 2007; Matschegewski et al., 2015; Ridge et al., 2015; Hasan et al., 2016; Irwin et al., 2016; Lin et al., 2018; Lin et al., 2019)
		*TFL*	Bo3g012730	(Okazaki et al., 2007; Duclos and Björkman, 2008; Siriwardana and Lamb, 2012; Schiessl et al., 2015; Branham et al., 2017; Leijten et al., 2018)
	DM C09@50.0	*FLC*	Bo(9g173400/9g173370)	(Okazaki et al., 2007; Matschegewski et al., 2015; Ridge et al., 2015; Hasan et al., 2016; Irwin et al., 2016; Lin et al., 2018; Lin et al., 2019)
		*GRF8*	Bo9g172070	(Wang et al., 2010)
		*SEP1*	Bo9g163790	(Varaud et al., 2011; Siriwardana and Lamb, 2012; Zhang et al., 2018)
		*CO*	Bo9g163730	(Osborn et al., 1997; Bohuon et al., 1998; Rae et al., 1999; Axelsson et al., 2001; Okazaki et al., 2007; Irwin et al., 2016; Leijten et al., 2018)
		*COL1*	Bo9g163720	(Lagercrantz et al., 2002)
**DF**	DF_C03@6.4	*FLC*	Bo3g024250	(Okazaki et al., 2007; Matschegewski et al., 2015; Ridge et al., 2015; Hasan et al., 2016; Irwin et al., 2016; Lin et al., 2018; Lin et al., 2019)
		*VIN3*	Bo3g019340	(Lin et al., 2005; Matschegewski et al., 2015; Ridge et al., 2015; Shea et al., 2018)
	DF_C09@50	*FLC*	Bo(9g173400/9g173370)	(Okazaki et al., 2007; Matschegewski et al., 2015; Ridge et al., 2015; Hasan et al., 2016; Irwin et al., 2016; Lin et al., 2018; Lin et al., 2019)
		*GRF8*	Bo9g172070	(Wang et al., 2010)
		*SEP1*	Bo9g163790	(Varaud et al., 2011; Siriwardana and Lamb, 2012; Zhang et al., 2018)
		*CO*	Bo9g163730	(Osborn et al., 1997; Bohuon et al., 1998; Rae et al., 1999; Axelsson et al., 2001; Okazaki et al., 2007; Irwin et al., 2016; Leijten et al., 2018)
		*COL1*	Bo9g163720	(Lagercrantz et al., 2002)

#### Biomass

In broccoli, higher yielding genotypes are preferred for commercial production and vegetative biomass is positively correlated with head biomass (Lin et al., 2013). In a F_2_ broccoli × broccoli population, Lin et al. (2013) identified biomass QTL on linkage groups C1, C5, C8, and C9, although the authors did not identify candidates associated with these loci. In a GWA study of seedling vigor in *B. napus*, the vigor candidates *Bna.SCO1*, *Bna.ARR4*, and *Bna.ATE1* were identified by Hatzig et al. (2015), although no homologous candidates were located within the BolTBDH VG BCIs. A GWA and transcriptome analysis in *B. napus* by Lu et al. (2017) identified two yield-related candidates: *BnaA05g29680D* and *BnaC04g42030D*. These candidates were not identified in BolTBDH biomass QTL, although one ortholog of *BnaA05g29680D* (Bo5g139830) was identified adjacent to the Bio_5_ hotspot.

The homologous candidates *BRC1* (Bo1g117490 and Bo5g117410) is closely related to *TEOSINTE BRANCHED1*, and is a putative transcription factor involved in arresting axillary bud development and limiting axillary bud growth (Muntha et al., 2018) and was identified within Bio_5_. Additional homologous VG candidates involved in growth and growth regulation were identified (**Table 6**).

#### Leaf Morphology

Variation in leaf morphology is useful to improve and develop novel market classes of *B. oleracea* leafy greens. Lan and Paterson (2000) identified robust QTL associated with leaf lamina width on C01 and C07, although they did not identify likely candidates. Previous studies have identified leaf-apex QTL on LGO1 and LGO3 (cauliflower × Brussels sprouts) (Sebastian et al., 2002) and C06 and C07 (broccoli × broccoli) (Walley et al., 2012). Li et al. (2009) identified the candidate *BrAS1* involved in leaf lamina width. The candidates *AtLUG*, *AtWOX1*, and *AtAN3* were shown by Zhang et al. (2019) to be involved in leaf blade outgrowth although none of these candidates were identified in LA BCI within BolTBDH. In *Arabidopsis*, *gif1* mutants exhibit a longer, narrow leaf phenotype (Shimano et al., 2018) and a *GIF1* homolog (Bo7g093130) collocated within LA_C07@36.6 QTL.

In a QTL-seq analysis of ornamental kale, Ren et al. (2019) identified a lobed-leaf candidate *BoLl* to C09 (38.82–40.12 Mb), although *BoLl* did not collocate with BolTBDH LM QTL. Ni et al. (2017) transformed *BnLMI1* into *Arabidopsis* producing serrated leaf margins, similar in appearance to the serrated leaf margin phenotype observed in BolTBDH. In BolTBDH, a *BoLMI1* ortholog (Bo3g002560) collocated closely with Lea_3_ in agreement with Ni et al. (2017).

Alterations in cuticular wax alters herbivore behavior and may confer broad resistance (Branham and Farnham, 2017). Leaf color in *B. oleracea* is strongly affected by cuticle wax content and is likely responsible for the variation in blue-green matte and dark-green glossy leaf appearance observed in the BolTBDH population. Xu et al. (2019) conducted fine mapping of the cuticular wax synthesis gene *BoWax1* controlling the glossy trait in a F_2_ cabbage population although this candidate was not identified in BolTBDH LC BCI. In another cabbage population segregating for the glossy leaves, Zhu et al. (2019) mapped a non-wax glossy *NWGL* locus to a 99 kb interval in C08. Neither *BoWax1* or the *NWGL* locus collocated with the LC QTL identified in BolTBDH. Lee et al. (2015b) analyzed expression of wax synthesis candidates and determined that the homologous candidates *LACS1*, *KCS1*, *KCR1*, *ECR*, *CER3*, and *MAH1* were differentially expressed in broccoli lines with elevated cuticular wax levels. Of these candidates, only *MAH1* homologs collocated with LC_C09@15.1 and the BCI included five *MAH1* copies: Bo9g053360, Bo9g053340, Bo9g053260, Bo9g053220, Bo9g053170. *MAH1* encodes CYP96A15, a midchain alkane hydroxylase, involved in cuticular wax biosynthesis (Greer et al., 2007).

#### Bud Morphology

*B. oleracea* flower petals are typically white, but a dominant mutation of *BoCCD4* implicated in a yellow-flower phenotype *via* inactivation of a carotenoid-degrading enzyme has been previously described (Zhang et al., 2015; Han et al., 2019). In BolTBDH, a single flower color QTL was identified (FC_C03@55.7) and *BoCCD4* (Bo3g158650; C03:56.61Mb) was located < 1 Mbp from this LOD peak, in agreement with (Zhang et al., 2015). Interestingly, 77.2% of BolTBDH lines exhibited a white flower phenotype, an unexpected result given single locus control in the MQM trait model FC ∼ 3@55.7, however the segregation distortion observed at this locus (*f* = 0.76 P_1_) is consistent with this result (**Figure 3B**).

Certain bud morphology defects contribute to a reduction in head quality, often rendering broccoli heads unmarketable. Hooked sepals are commonly encountered in broccoli hybrids, resulting in a non-uniform crown surface and a reduced ability to shed water. In *B. rapa*, Zhang et al. (2018) identified loss-of-function *BrAP2* alleles with sepal defects similar to the hooked sepal phenotype observed in BolTBDH. In BolTBDH, the optimal MQM model for hooked sepals (SH ∼ 1@2.1 + [1@0.2 × 1@20.2]) co-localized with the bud morphology hotspot Bud_1_. Bud_1_ QTL include the homologous candidates *AP2* (Bo1g004960), in agreement with Zhang et al. (2018). Further evaluation of *AP2* (Bo1g004960) homologs and other floral developmental candidates (**Table 6**) in the Bud_1_ hotspot may prove useful for improvement of bud morphology traits.

Small flower buds are preferred in broccoli, but heat-tolerant germplasm typically exhibits larger flower buds. MQM modeling of bead size in BolTHDH resulted in a complex model trait model: BS ∼ Bud_1_ + 3@36.8 + Bio_5_ + 6@3.8 + [Bud_1_ × Bio_5_] + [Bio_5_ × 6@3.9], suggesting complex genetic control of this trait.

Unequal-sized flower buds are a common horticultural defect in broccoli and bud uniformity requires an arrest of enlargement of older buds until younger buds reach an equivalent size, requiring complex coordination (Roeder et al., 2010). Lin et al. (2018) identified a QTL within CO6 (*qCQ-6*) associated with a reduction in uneven-sized flower buds and identified *PAN* and the most probable candidate within this interval, exhibiting strongly differential expression in floral bud at harvest stage. In the BolTBDH model of flower bud uniformity (BU ∼ Lea_3_ + 4@51.5), the *PAN* homolog Bo6g107140 was not harbored within the identified BU QTL.

#### Head Quality

For heading quality traits, the hotspot HQ_9_ was remarkably pronounced within MQM analysis of BolTBDH. HQ_9_ is syntenic with the telomeric region of the short arm of *Arabidopsis* Chr5 (O’neill and Bancroft, 2000), and this region has previously been shown to carry the homologs of the key flowering-time and vernalization-response genes *TLF2*, *COL1*, *CO*, and *FLC* (Osborn et al., 1997; Lagercrantz et al., 2002; Lin et al., 2005; Okazaki et al., 2007; Uptmoor et al., 2008; Iniguez-Luy et al., 2009; Hasan et al., 2016; Shea et al., 2018). Razi et al. (2008) mapped *BoFLC1* to the end of C09, and homology searches in the BOLv.2 genome indicated that two *FLC* copies (Bo9g173370 and Bo9g173400) appear to be harbored within the HQ_9_ hotspot. These putative *BoFLC1* copies are located ∼22 Kbp apart and may have been considered as a single copy in previous studies. In broccoli and cauliflower, this region has been implicated in vernalization requirement response (Bohuon et al., 1998; Rae et al., 1999), heat-tolerance (Branham et al., 2017), temperature dependent curd induction (Hasan et al., 2016), variation in heading response to temperature (Lin et al., 2019) and as a target for domestication due to a sharp increase in linkage disequilibrium when comparing improved broccoli and landrace broccoli genotypes (Stansell et al., 2018). Here, we identified HQ_9_ as containing disproportionately many key heading-quality broccoli QTL: BU_C04@51.5, BR_C09@49.5, HC_C09@48.8, HU_C09@48.8, HE_C09@47.7, and OQ_C09@49.5, as well as two phenology QTL, DM_C09@50.0 and DF_C09@50.0.

Broccoli is characterized by strong suppression of bract elongation in the inflorescence and the “leaf in curd” bracting phenotype has been previously linked to high temperature stress (>22°C) (Booij and Struik, 1990). Kop et al. (2003) identified a role *Boap1-a* in bracting suppression within broccoli and cauliflower heads, although the authors suggested that additional candidates may be involved in bract development, e.g., *BoFUL*. Neither *AP1* (Bo2g062650, Bo6g095760, Bo6g095760, Bo6g108600), or *FUL* (Bo2g161210, Bo7g098190, Bo9g014400) homologs cosegregated with bracting loci identified within the BolTBDH MQM bracting model.

Compact broccoli heads are less susceptible to damage and are more efficient to ship. In broccoli, the degree of head compactness is a consequence of a short rachis at a large angle. In a F_2_ *B. oleracea* cross, Lan and Paterson (2000) identified a C04 QTL (EW4D04w+7) likely implicated in curd density. A more complex head compactness model was observed in BolTBDH (HC ∼ HC_6@23.0 + HQ_9_ + [HQ_9_ × 3@53.6] + [HQ_9_ × 5@1.4] + [HQ_8_ × 3@53.6]).

Along with head compactness, head diameter at maturity is an important element of high yielding cultivars. In three *B. oleracea* F_2_ populations, Lan and Paterson (2000) identified 14 curd width QTL on chromosomes C01, C03, C4, C5, C7, C8, and C9. Within a broccoli × broccoli mapping population, Walley et al. (2012) identified QTL for head diameter on linkage groups C2, C4, C6, C7, and C9. Head diameter within BolTBDH was captured by the MQM model HD ∼ HD_C05 @2.9 + HD_C07@43.6/46.5 + [HD_C05@2.9 × HD_C07@43.6/46.5] + [Bud_1_ × HD_C05@2.9].

Head extension above lead rosette is a useful trait in broccoli by reducing labor during harvest. Alternatively, late stem elongation may be a useful to protect the head during growth. The MQM model of head extension is complex: HE ∼ HQ_9_ + HQ_8_ + C06@38.5 + [HQ_9_ × 6@38.5] + [HQ_9_ × 2@5.5].

Convex head shape is an important trait in broccoli by allowing the crown to shed water, thereby reducing disease incidence and exhibits complex control HC ∼ HQ_8_ + C02@41.7 + C09@37.1 + [HQ_8_ x HQ_9_] + [2@41.7 × 9@37.1].

As expected, overall broccoli heading quality is the most complex of the head quality traits, best captured by the MQM model: OQ ∼ 3@57.0 + HQ_4_ + HQ_8_ + HQ_9_ + [HQ_8_ × 3@53.6] + [HQ_8_ × HQ_9_] + [HQ_9_ × 5@1.3]. A simple control model of the broccoli heading-phenotype due to quantitative control of heading quality traits may be excluded given the MQM model determined in BolTBDH. Additionally, a constrictive-conditional model explaining the broccoli heading-phenotype seems less probable given the relative independence of heading quality traits. For example, the MQM models for HS and HU share HQ_9_, but HS does not share HQ_4_ with the overall heading quality model, nor does HU share HQ_8_ with the overall heading quality model (**Figure S4**). The arrested-meristem broccoli heading-phenotype is best explained by a pleiotropic model — where a small number of genes within HQ_9_ are implicated in multiple heading quality traits, and these HQ_9_ genes exhibit important epistatic interactions with other heading quality loci.

#### Phenology

In a previous study of days to flowering within a different *albogabra* × *italica* mapping population, Bohuon et al. (1998) identified QTL on C02, C03, C05, and C09. In a QTL-seq analysis of broccoli × cabbage, Shu et al. (2018) identified three flowering time regions: C02@0.9–2.9 Mb, C03@1.8–20 Mb, and C06@5.0–5.6 Mb. Only the C03@1.8–20 Mb region collocated with the DF QTL identified in BolTBDH (DF_C03@6.4). Using a relative expression approach, Abuyusuf et al. (2019) report sequence based variations in *BoFLC1.C9* (C09:51.0–51.0 Mb) implicated in early and late flowering cabbage genotypes, in agreement with the DM_C09@50.0 and DF_C09@50.0 QTL identified in BolTBDH. In a study of curd initiation in DH cauliflower, Hasan et al. (2016) identified a temperature-dependent time to curd induction QTL on C09 at 49.4 Mb, closely collocating with DM_C09@50.0 and DF_C09@50.0 and identified additional days to flowering QTL on C04, C05, C06, and C07. Within BolTBDH, days to head maturity and flowering appear to be each strongly influenced by two hotspots HQ_9_ × PHE_3_ given by the QTL pairs (DM C03_6.4 and DF_C03@6.4) and (DM_C09@49.7 and DF_C09@49.7) and these pairs exhibit strong epistatic effects ([DM_3@6.4 × DM_9@50.0]; p_fv1_ << 0.01) and [DM_C09@50.0 × DF_C09@50.0] (pfv1 << 0.01). One additional DM epistatic effect was detected ([HQ9 x 7@DM_42.0]; p_fv1_ = 0.023). Interestingly, HQ_9_ collocates with the region of strongest genome-wide segregation distortion (P_1_ allele: *f* = 0.76). If late-flowering DHs were underrepresented during tissue culture or seed regeneration, the P_1_ allele and surrounding region would exhibit segregation enrichment.

Lan and Paterson (2000) identified 15 “days from budding to flowering” QTL, analogous to the holding ability trait measured in BolTHDH population. In BolTBDH, “days from head maturity trait to flowering” was best explained by a complex MQM model: HA ∼ 5@4.0 + 6@25.9 + 7@41.2 + [9@9.5 × 9@10.7] + [5@36.6 × 5@37.3] and no likely homologous candidates were identified.

### Conclusions

Evaluation of the BolTBDH population provides new insights into key genomic regions and developmental candidates that define heading broccoli by identifying essential QTL implicated in these phenotypic outcomes. These results support a pleiotropic model of a heading broccoli phenotype. This work demonstrates several key genomic hotspots as essential for the phenotypes observed within this study, and these QTL and markers may prove useful for future marker-assisted breeding efforts. The phenomic and genomic dataset provided herein may be used for additional mapping studies and be integrated with previous work (e.g. metabolic and pathogen resistance studies).

## Data Availability Statement

The datasets used for this study are included in *Stansell_2019_Supp_Data.zip*. The code used for this study is available at:

- https://github.com/zacharystansell/BolTBDH
- https://github.com/zacharystansell/ratervar

## Author Contributions

ZS and TB conducted experimental design, ZS conducted phenotyping and statistical analyses. MF provided experimental germplasm. All authors wrote the manuscript.

## Funding

This work is supported by Specialty Crop Research Initiative grant no. 2016-51181-25402 from the USDA National Institute of Food and Agriculture.

## Conflict of Interest

The authors declare that the research was conducted in the absence of any commercial or financial relationships that could be construed as a potential conflict of interest.
