# Supplementary material for: Complex Horticultural Quality Traits in Broccoli Are Illuminated by Evaluation of the Immortal BolTBDH Mapping Population
Source: Front Plant Sci. 2019 Sep 18;10:1104. doi: 10.3389/fpls.2019.01104 (PMC6759917; doi:10.3389/fpls.2019.01104)
Supplement: Supplementary file 1 [file DataSheet_1.pdf]

# Supplementary Material

## 1 SUPPLEMENTARY DATA

- Supplemental Data FILE 1: py1.csv; $Y_{12}$ cleaned genotype and phenotype data in *.csvr format [1.1 MB] for r/qtl
- Supplemental Data FILE 2: py1y2.csv; $Y_1$ cleaned genotype and phenotype data in *.csvr format [1.0 MB] for r/qtl
- Supplemental Data FILE 3: py2.csv; $Y_2$ cleaned genotype and phenotype data in *.csvr format [1.0 MB] for r/qtl
- Supplemental Data FILE 4: all.pheno.csv; $Y_{12}$ phenotype data in *.csv format [37.8 kB]
- Supplemental Data FILE 5: p17.pheno.csv; $Y_1$ phenotype data in *.csv format [27.3 kB]
- Supplemental Data FILE 6: p18.pheno.csv; $Y_2$ phenotype data in *.csv format [26.2 kB]
- Supplemental Data FILE 7: candidate.intersection.csv; Intersection of BCI and candidates in *.csv format [53.9 kB]

## 2 SUPPLEMENTARY TABLES AND FIGURES

| trait | $R^2$ | differences | lower | upper |
|---|---|---|---|---|
| HU | 0.2466 | A-B | 0.2122 | 0.2759 |
| HC | 0.2131 | A-B | 0.1862 | 0.2362 |
| HS | 0.1685 | C-D | 0.1425 | 0.1901 |
| BR | 0.1515 | C-D | 0.1252 | 0.1753 |
| BU | 0.0388 | E-I | 0.0229 | 0.0628 |
| DF | 0.0292 | E-J | 0.0194 | 0.0428 |
| HD | 0.0284 | E-L | 0.0143 | 0.0500 |
| DM | 0.0281 | F-J | 0.0188 | 0.0399 |
| HE | 0.0267 | E-K | 0.0152 | 0.0443 |
| LT | 0.0195 | G-L | 0.0123 | 0.0316 |
| LC | 0.0161 | G-N | 0.0075 | 0.0290 |
| MS | 0.0136 | I-N | 0.0059 | 0.0264 |
| VG | 0.0092 | J-O | 0.0035 | 0.0202 |
| LM | 0.0057 | L-P | 0.0017 | 0.0145 |
| LA | 0.0034 | L-P | 0.0007 | 0.0123 |
| BS | 0.0017 | N-P | 0.0010 | 0.0067 |

**Table S1.** Traits used in relative importance analysis (RIA) results partitioning overall heading-quality model $R^2$ using method “lmg”, differences are conducted as separation of means; lower and upper are 95% confidence intervals estimated from 1000 bootstrap replications.

| chr | n.mar | length | ave.spacing | max.spacing |
|---|---|---|---|---|
| C01 | 106 | 86.89 | 0.83 | 2.86 |
| C02 | 192 | 131.75 | 0.69 | 8.07 |
| C03 | 283 | 139.71 | 0.50 | 2.87 |
| C04 | 238 | 123.09 | 0.52 | 5.16 |
| C05 | 212 | 170.25 | 0.81 | 16.67 |
| C06 | 196 | 78.13 | 0.40 | 6.90 |
| C07 | 240 | 93.14 | 0.39 | 3.75 |
| C08 | 175 | 74.30 | 0.43 | 2.26 |
| C09 | 239 | 163.56 | 0.69 | 12.17 |
| total | 1881 | 1060.81 | 0.57 | 16.67 |

**Table S2.** BolTBDH genetic mapping summary statistics disaggregated by chromosome by number of markers (**n.mar**), length (**length**; cM), average spacing (**avera.spacing**; cM/marker), and maximum spacing (**max.spacing**; cM/marker).

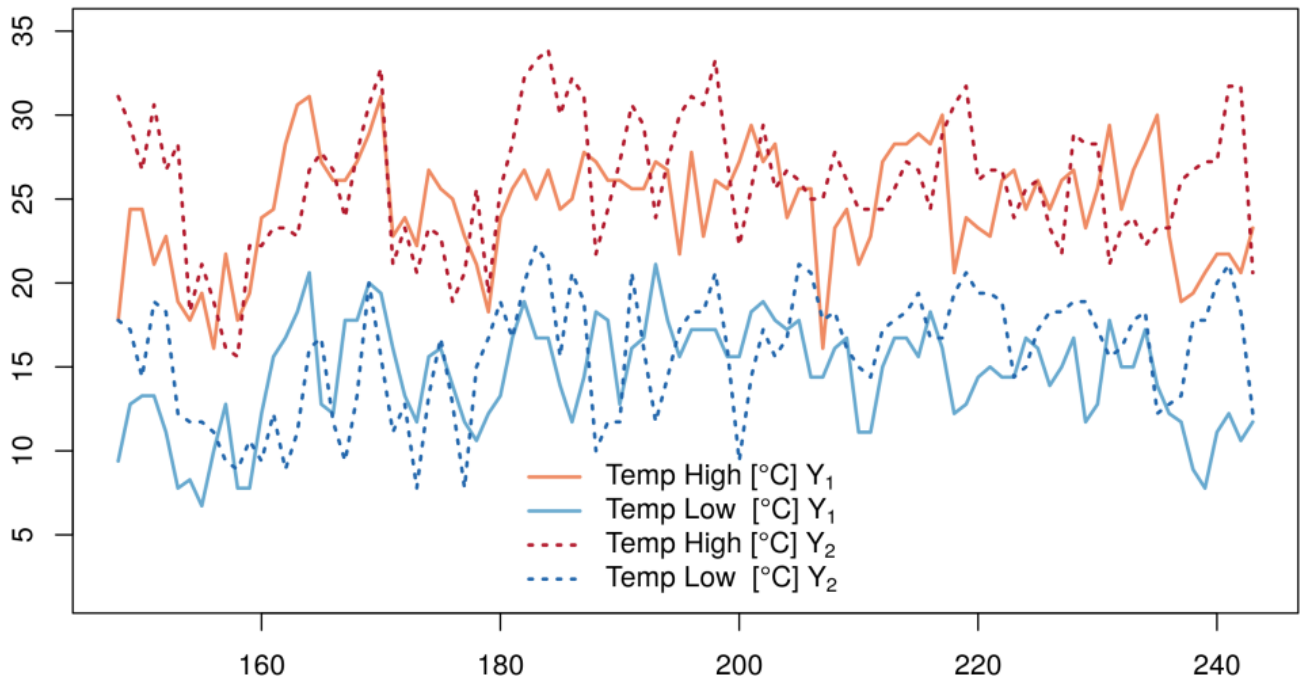


**Figure S1.** Year 1 ($Y_1$) and year 2 ($Y_2$) daily high and low temperatures (X-axis [Julian Date]; Y-axis [°C]).

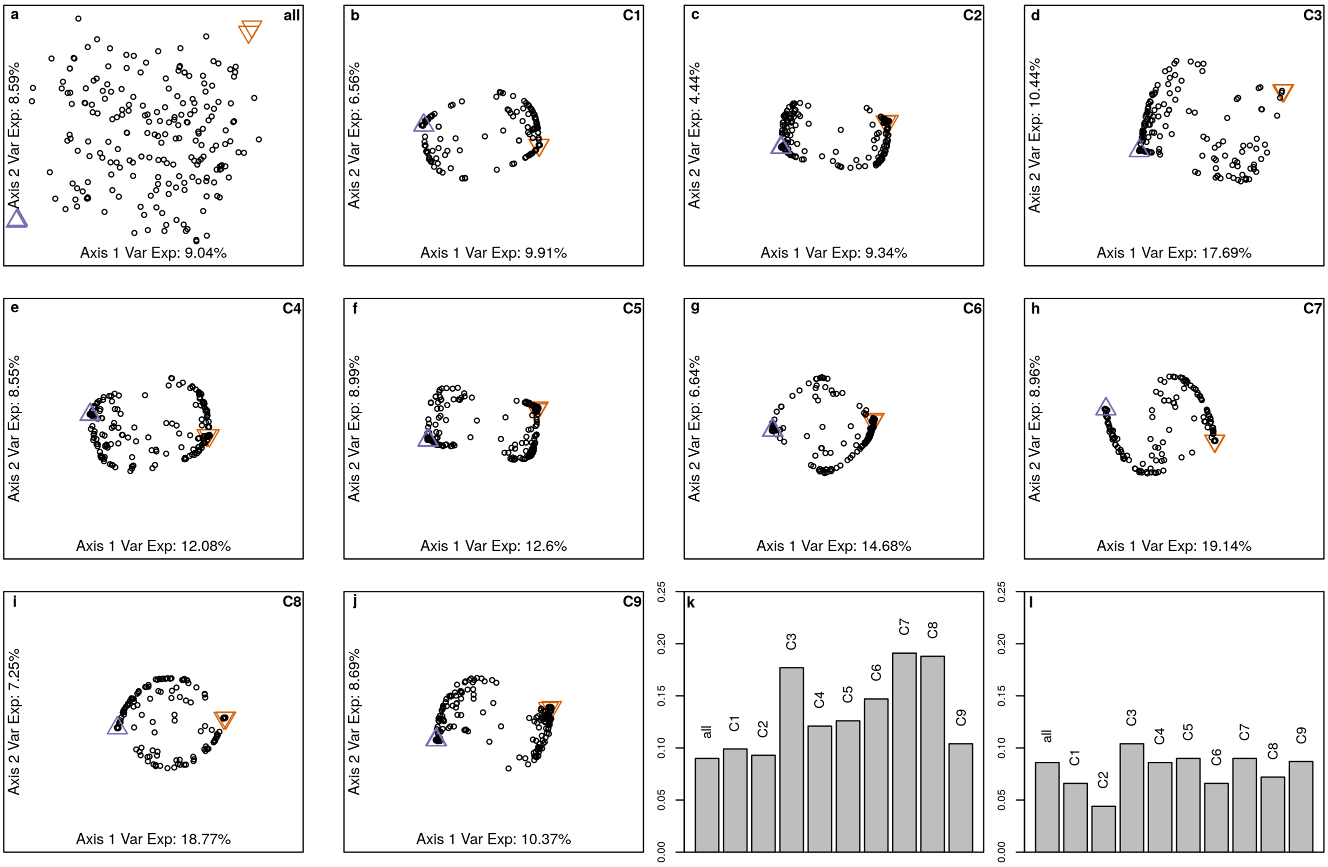


**Figure S2.** Principal component analysis of all GBS SNPs by line: all SNPs pooled genome-wide (**A**) and by chromosome(**B-J**). Parental lines 'Early Big' and 'TO1000' are represented by orange and purple triangles. Percent variation PCA1 (**J**) and PCA2 (**K**) are compared. Chromosomes exhibiting stronger segregation distortion signals typically displayed higher PCA1 values.

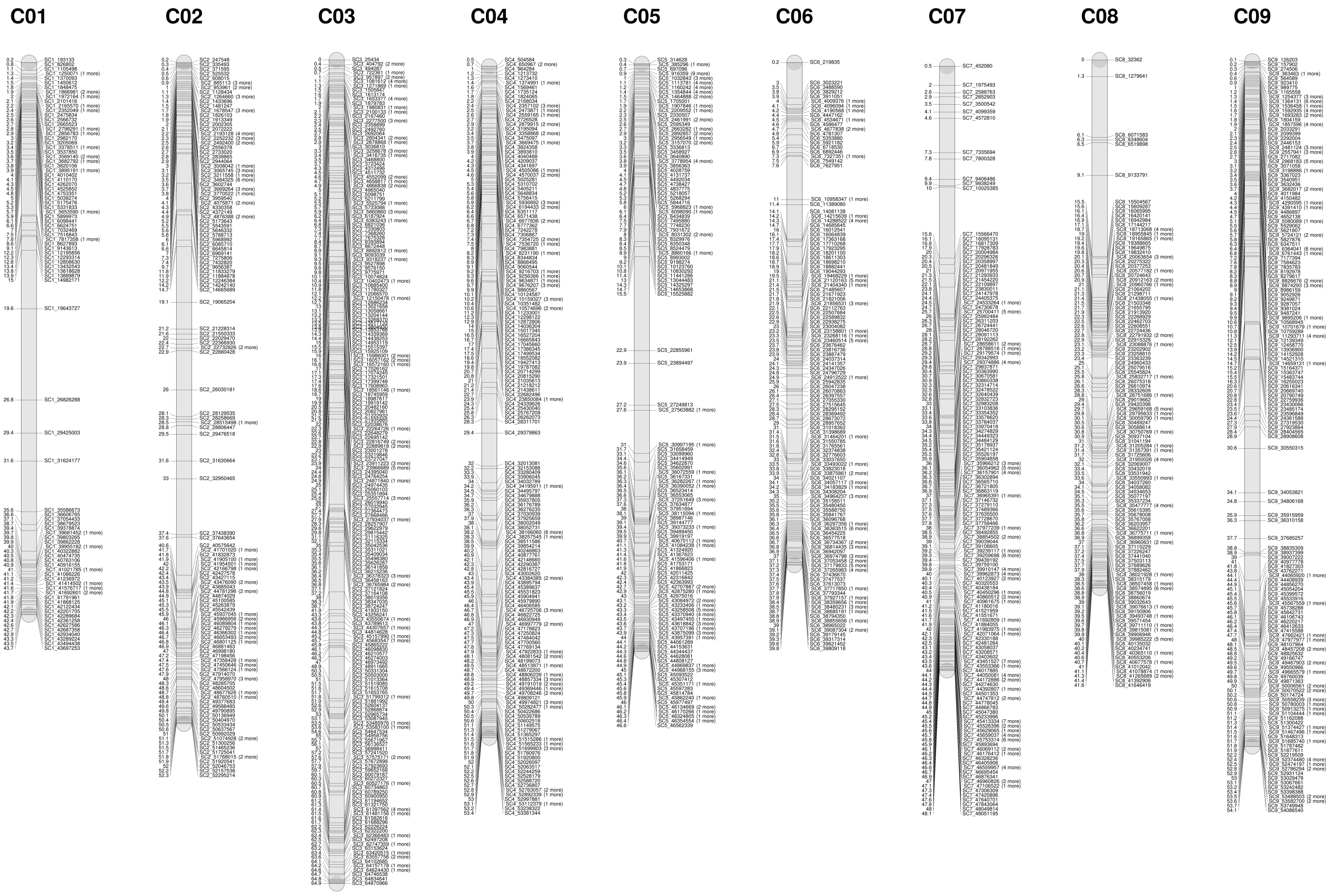


**Figure S3.** Genetic map of chromosomes C01-C09 constructed with all 1881 BolTBDH markers. Marker locations (Mbp) printed on the left and marker names printed on the right of each chromosome.

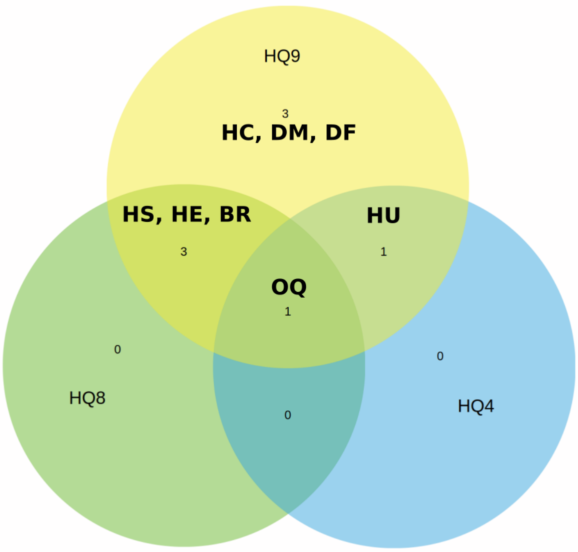


**Figure S4.** Colocation of heading quality and phenology QTL with MQM broccoli quality model given by $\mathbf{OQ} \sim 3@57.0 + \mathbf{HQ_4} + \mathbf{HQ_8} + \mathbf{HQ_9} + [\mathbf{HQ_8}\text{x}\mathbf{HQ_9}] + [\mathbf{HQ_8}3@53.6] + [\mathbf{HQ_9}\text{x}5@1.3]$.
